# Dynamic contact network between ribosomal subunits enables rapid large-scale rotation during spontaneous translocation

**DOI:** 10.1093/nar/gkv649

**Published:** 2015-06-24

**Authors:** Lars V. Bock, Christian Blau, Andrea C. Vaiana, Helmut Grubmüller

**Affiliations:** Department of Theoretical and Computational Biophysics, Max Planck Institute for Biophysical Chemistry, Am Fassberg 11, 37077 Göttingen, Germany

## Abstract

During ribosomal translation, the two ribosomal subunits remain associated through intersubunit bridges, despite rapid large-scale intersubunit rotation. The absence of large barriers hindering rotation is a prerequisite for rapid rotation. Here, we investigate how such a flat free-energy landscape is achieved, in particular considering the large shifts the bridges undergo at the periphery. The dynamics and energetics of the intersubunit contact network are studied using molecular dynamics simulations of the prokaryotic ribosome in intermediate states of spontaneous translocation. Based on observed occupancies of intersubunit contacts, residues were grouped into clusters. In addition to the central contact clusters, peripheral clusters were found to maintain strong steady interactions by changing contacts in the course of rotation. The peripheral B1 bridges are stabilized by a changing contact pattern of charged residues that adapts to the rotational state. In contrast, steady strong interactions of the B4 bridge are ensured by the flexible helix H34 following the movement of protein S15. The tRNAs which span the subunits contribute to the intersubunit binding enthalpy to an almost constant degree, despite their different positions in the ribosome. These mechanisms keep the intersubunit interaction strong and steady during rotation, thereby preventing dissociation and enabling rapid rotation.

## INTRODUCTION

The ribosome is a macromolecular RNA–protein complex synthesizing proteins in the cell by translating messenger RNA (mRNA). The ribosome (70S in prokaryotes) consists of two subunits; the small (30S) subunit mediates base pairing between mRNA and transfer RNAs (tRNAs) and the large (50S) subunit catalyzes peptide bond formation. The tRNAs, which deliver amino acids to the growing peptide chain, bind to three sites on both ribosomal subunits, the aminoacyl (A), the peptidyl (P) and the exit (E) site. After peptide bond formation, in a process called translocation, the peptidyl-tRNA moves from the classical pre-translocation A site through a hybrid A/P site (bound to 30S A and 50S P sites, respectively) to the classical post-translocation P site. The deacylated tRNA moves from the classical P site first to the hybrid P/E and then to the E site, where it finally dissociates from the ribosome. GTP hydrolysis by elongation factor G (EF-G) drives translocation (1), but in the absence of the factor, tRNAs spontaneously translocate albeit at slower rates (2,3).

Translocation is accompanied by large-scale conformational changes of the ribosome, including intersubunit rotation (4–20) and L1 stalk movement (5,7,8,21–26). Rotation of the 30S body relative to the 50S subunit (body rotation) is required for translocation (27) and during spontaneous translocation ranges from −3 to 16° (24,25). Recently, single molecule Förster Resonance Energy Transfer (smFRET) experiments suggested a hyper-rotated state of the ribosome with an estimated rotation of 22° in the presence of structured mRNA downstream of the ribosome's mRNA tunnel (28). The rotation of the 30S head domain relative to the 30S body (head swiveling) has been suggested to be directly involved in the rate-limiting step of translocation, the movement of the tRNAs from A and P to P and E sites on the 30S subunit (10,15,17,20,29–31). During all these conformational changes, the affinity between the two subunits has to be tightly controlled—strong enough to allow the subunits to assemble upon initiation and to remain associated during translation (32) and weak enough to allow dissociation of ribosomes after termination (33).

Rapid spontaneous rotation has been observed in smFRET experiments (34) and in molecular dynamics (MD) simulations (25) implying an absence of large free-energy barriers hindering intersubunit rotation. This ‘flatness’ of the rotation free-energy landscape requires similar affinities for different intersubunit rotation angles, because high affinity for specific angles and low affinity for intermediate angles would lead to free-energy barriers. Indeed, the ribosome shows almost no preference for specific rotation angles at room temperature (24).

Here, we address the riddle of how the complex interaction network between the subunits ensures stability of the ribosome despite the large relative shifts, which the contact surfaces of the subunits undergo during intersubunit rotation. Further, we study the main structural determinants for achieving sufficiently constant affinity between the subunits for different rotation angles, a prerequisite for rapid rotation.

**Figure 1.**
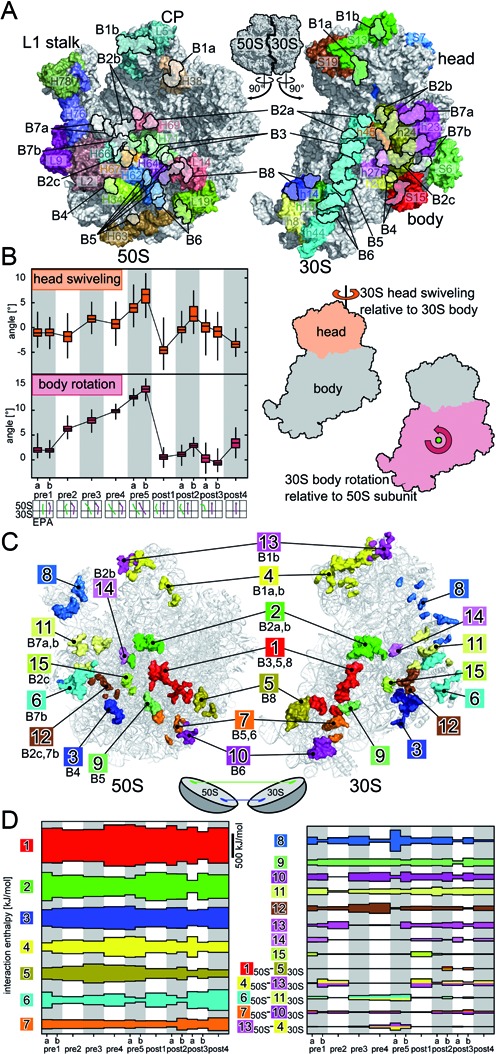
(**A**) Intersubunit surface of large (50S) and small subunit (30S) with colored rRNA helices (H for 50S, h for 30S) and proteins (L for 50S, S for 30S) involved in intersubunit contacts. The black outlines depict residues involved in intersubunit bridges as defined by Gao *et al*. (37). Positions of the L1 stalk, central protuberance (CP), 30S head and body are shown. (**B**) Box plot of head swiveling and body rotation angles observed in simulations spontaneous translocation intermediates (left). Positions of rotation axes for head swiveling (31) and body rotation (25). (**C**) Clusters of residues contributing to intersubunit contacts (colored surfaces) between 30S and 50S subunits (gray); Clusters are labeled by numbers and by the conventional bridge names (37). (**D**) Interaction enthalpies between 30S and 50S parts of contact clusters for different tRNA translocation intermediates.

The two ribosomal subunits are associated through 12 intersubunit bridges (B1a/b, B2a/b,c, B3, B4, B5, B6, B7a/b, B8, see Figure 1a), groups of residues interacting across the subunits, which have been identified by cryo-electron microscopy (cryo-EM) (4,5,30,35–38) and X-ray-crystallography studies (11,29,39–41). Five bridges are solely composed of contacts between 23S rRNA nucleotides (50S subunit) and 16S rRNA nucleotides (30S subunit): the central B2a/b/c and B3 bridges and the peripheral B7a bridge (37). These central RNA–RNA bridges, whose conformations change little during intersubunit rotation, have been suggested to be responsible for maintaining 70S stability (37) and to serve as anchoring patches for intersubunit rotation (42). Other bridges involve additional 50S (B5, B6) or 30S protein residues (B4). Further, bridge B1a is formed of contacts between 23S rRNA nucleotides and 30S protein residues, whereas bridges B7b and B8 are composed of 16S rRNA nucleotides and 50S protein residues. For bridges B1a, B2a/b/c, B3, B4 and B7a, 23S rRNA mutations that affect 70S formation were reported (43–46). On the 30S subunit, modification of 16S rRNA revealed that B2a/b, B3, B5 and B7a are essential for subunit association (47). These studies show that the stability of the ribosome is determined and controlled by a large network of intersubunit interactions.

The B1 bridges are the only ones located on the 30S head (Figure 1a) and were found to change conformation during intersubunit rotation (4,5,11,29,33,37,38,48). B1b, the only protein–protein bridge, is formed by contacts between proteins L5 and S13 (50S/30S proteins and 23S/16S rRNA helices are labeled L/S and H/h, respectively). Ribosomes lacking S13 show an increased rate of tRNA translocation, suggesting that the L5-S13 contacts stabilize pre-translocation states (49). Truncation of helix H38, which is involved in bridge B1a, might lead to an increased rate of translocation as well (50), but this issue remains controversial (44,51).

The 23S rRNA helix H34 contacts protein S15 on the 30S body forming intersubunit bridge B4. In a crystal structure of a ribosome with a ∼9° body rotation, H34 was seen to bend compared to the non-rotated conformation (11). This bending allowed H34 to maintain the bridge B4 contacts with S15, despite the shift of S15 relative to H34 induced by the rotation. Body rotations of up to 16° were observed by cryo-EM (24), raising the question if the bridge B4 contacts rupture at high rotation angles.

Mutations disrupting bridge B1a located on the 30S head, as well as 30S body bridges B4, B7a and B8 result in increased EF-G driven forward as well as spontaneous back translocation rates (52). This finding suggests that both 30S body rotation and 30S head swiveling are involved in unlocking, the rate-limiting step of translocation (53).

Besides 70S assembly and tRNA translocation, intersubunit bridges are also involved in initiation (54), decoding (55) and ribosome recycling (11,33). The central bridge B2a plays a crucial role in all of these processes. During 30S initiation complex formation, the binding of initiation factor 3 (IF3) to the 30S subunit impairs the formation of bridge B2a, thus hindering premature subunit assembly (54). After translation termination, binding of the ribosome recycling factor (RRF) results in a rotated ribosome containing P/E tRNA and a change in B1 bridge conformation (11,33). RRF in complex with EF-G and the 50S subunit, representing the complex after subunit dissociation, overlaps with bridges B2a and B3 suggesting that the breaking of these bridges leads to dissociation (33). Helix H69, which participates in forming bridges B2a and B2b, shifts upon binding of RRF to the 50S subunit (56), indicating the importance of the dynamics of intersubunit bridges for ribosomal stability. Bridge B2a is also a target for antibiotics, such as viomycin (57) and neomycin (58), which trap the ribosome in certain rotation intermediates, thereby hindering translocation as well as factor binding and consequently protein synthesis (34,58,59).

In addition to intersubunit bridges, tRNAs, which bind to the 30S and 50S subunits, markedly contribute to the stabilization of subunit association (32,60). Apart from stabilization of the complex, the binding of the tRNAs to different binding sites might also perturb the rotation free-energy landscape.

To address the question of how steady and strong interactions can be maintained despite the large relative shifts of the surfaces during rotation, we analyzed the dynamics and energetics of the entire intersubunit contact network observed in MD simulations of 13 intermediate states of spontaneous tRNA translocation (25). These 13 states comprise 7 pre-translocation states (pre1a–pre5b) with gradually increasing body rotation and 6 post-translocation states (post1–post4). Previously, slow transition rates were found for 30S head swiveling and body rotation between early pre-states (pre1a–pre2) and late pre-states (pre3–pre5b) as well as between late pre- and post-states. To describe the intersubunit contact network, residues were grouped into clusters based on contacts observed in the simulations. In particular, we investigated the mechanisms by which bridges B1a, B1b and B4 maintain steady interaction while being subject to large shifts of their 30S residues relative to their 50S residues.

## MATERIALS AND METHODS

### Molecular dynamics simulations

Initial ribosome models including mRNA and tRNAs based on crystal structures (9,61) were refined against 13 cryo-EM reconstructions of intermediate states of spontaneous tRNA translocation (24), as described in our previous work (25). Subsequently, 100-ns all-atom MD simulations were started from all 13 refined structures. All simulations were carried out in explicit solvent with the software package GROMACS 4.5 (62), using the amber99sb forcefield (63), the SPC/E water model (64) and K^+^Cl^−^ ion parameters from Joung *et al*. (65). The system setup and the MD protocol were applied as described earlier (25).

### 30S body rotation and head swiveling

Rotation angles around previously described axes for 30S head swiveling (31) and 30S body rotation (25) were calculated from all the trajectories.

### Clustering of intersubunit contacts

To describe the dynamics and energetics of the intersubunit interactions, residues involved in intersubunit contacts were clustered into groups. Two residues were considered to be in contact if the minimum distance between any two atoms of the respective residues was below 3 Å. Pairs of residues in contact in at least 1% of the frames from any trajectory were extracted using the program g_contacts (66) and used for further analysis.

In different states, different residues were involved in intersubunit contacts. We aimed at merging interconnected residues into groups, while still keeping groups that are not connected by stable contacts in at least one state separate.

For that purpose, in a first stage, the contacts with an occupancy of at least 30% were clustered according to the following four steps (see schematic in Supplementary Figures S1, S2 and Supplementary Methods):

- For each state all residues connected by intersubunit contacts in the corresponding simulation were put into one cluster (Supplementary Figure S1a). Hence, we obtain a different set of contact clusters for each state.
- To obtain a common set of clusters for all states, all clusters from the individual states were ordered according to the number of their residues (Supplementary Figure S1b). The new common set of clusters was defined as initially containing only the smallest cluster, i.e the cluster with the fewest residues, (Supplementary Figure S1c, cluster A). Iteratively this set of common clusters was extended by adding the next (larger) cluster that did not overlap with any of the new common clusters (Supplementary Figure S1d, clusters B, C, D).
- Each of the new common clusters was extended to include residues from all remaining clusters which overlap only with this new cluster (Supplementary Figure S1e, cluster D).
- Next, the residues of the remaining clusters were assigned to the closest new common cluster (Supplementary Figure S1e, clusters B, C). Here, the closeness of a residue to a cluster is defined as the sum of occupancies of the contacts this residue has with any residue in the cluster.

**Figure 2.**
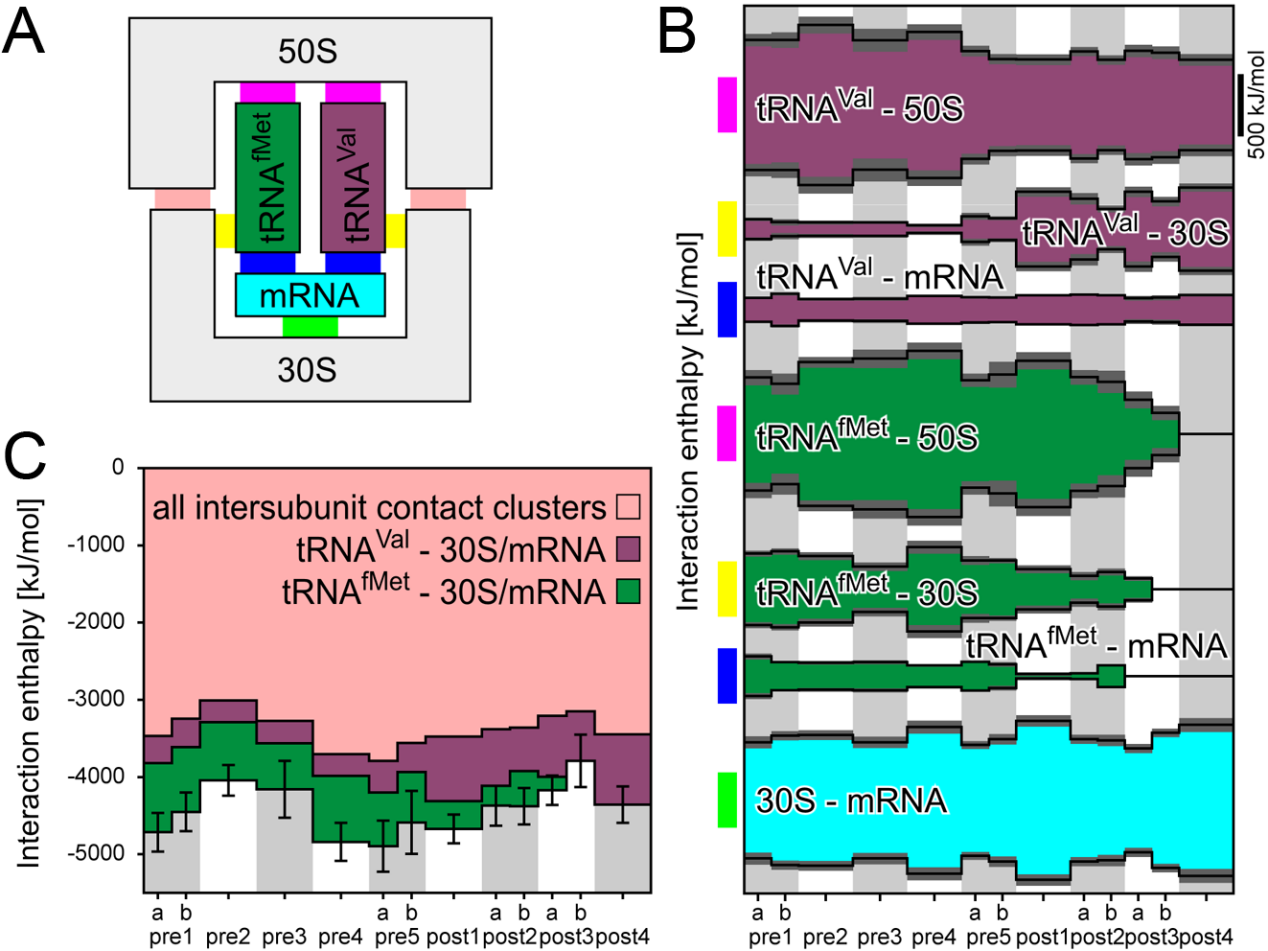
(**A**) Schematic of interactions between tRNAs and 50S, 30S subunits as well as the mRNA. (**B**) Interaction enthalpies for the interactions indicated in (A). Gray areas denote the standard deviation. (**C**) Comparison of the sum of all direct 30S–50S interaction enthalpies (light red, compare Figure 1d, with the enthalpic contributions of the tRNAs (magenta, green) to the intersubunit association. The black bars denote the standard deviations of the sum of these three contributions.

In the second stage, the residues involved only in contacts with an occupancy below 30% were iteratively sorted into the closest new clusters. The remaining six residues that did not interact with any of the residues in the new clusters were put into a separate group.

As a result, a set of 16 intersubunit contact clusters containing residues linked by mutual contacts between the subunits and one group of residues that contains residues which do not form contacts with these clusters were obtained.

### Interaction enthalpies

To estimate the contribution of each intersubunit contact cluster to the overall affinity, interaction enthalpies (the sum of electrostatic and van-der-Waals interactions between the 30S and the 50S part of the cluster) were calculated for each frame of each trajectory. The electrostatic and van-der-Waals interactions were calculated with the point charges and Lennard-Jones parameters from the amber99sb forcefield (63) using GROMACS (62). Next, for each cluster and state, the interaction enthalpy was averaged over all frames. One of the contact clusters and the group of residues not interacting with any cluster were not considered for further analysis, because the average enthalpy of the corresponding residues was found to be <1 % of the sum of all enthalpies in each state. This leaves a set of 15 intersubunit contact clusters.

When bound to the ribosome, the tRNAs are bridging the subunits. To estimate the contributions of the tRNAs to the ribosomal intersubunit interaction in the different binding sites, their interaction enthalpy with the 30S and 50S subunits, with the mRNA and the interaction enthalpy of the mRNA with the 30S were averaged for each translocation intermediate state.

We note that potential additional enthalpic interactions between the subunits mediated by ions and water molecules as well as entropic contributions are not accounted for.

Hydrogen bond energies were estimated as described (67) from donor–acceptor distances extracted using the program g_hbond (62).

### Restriction of contacts

The 30S parts of the intersubunit contact clusters shift relative to their 50S counterparts during intersubunit rotation. Some residues are only involved in a single contact throughout states, whereas other residues change their contact partners in different translocation intermediate states. To quantify to which extent residues change their contact partners, we have defined a level of contact restriction as follows:

First, the development of the residue–residue contact $\mathbf {c}^i$ over the 13 different states is described by a vector

\begin{equation*} \mathbf {c}^i=\left(c^i_1,c^i_2,\dots ,c^i_{13}\right), \end{equation*}

where $c_s^i \in (0,1)$ describes the presence (1) or absence (0) of the corresponding contact in state *s*. The contact $c_s^i$ was set to 1 if the contact occupancy was above 0.25 and 0 otherwise.

For each residue of the intersubunit contact clusters, all *n* contacts of this residue $\mathbf {c}^1,\dots ,\mathbf {c}^n$ were extracted. Next, for each pair of contacts $\mathbf {c}^i$ and $\mathbf {c}^j$ a restriction score

(1) \begin{eqnarray*} r\left(\mathbf {c}^i,\mathbf {c}^j\right)=\left\lbrace \begin{array}{l l}0 & \textrm {if } \mathbf {c}_s^i\cdot \mathbf {c}_s^j=0\\ 1 & \textrm {else } \end{array}\right. \end{eqnarray*}

was calculated. By this definition, the restriction score of two contacts is 1 if both contacts are present at the same time in any of the states. If the first contact is only present in states in which the second contact is absent and *vice versa*, the restriction score is 0.

The restriction score *R* of a certain residue is defined by the average of the restriction scores of all *n*(*n* − 1)/2 unequal pairs of contacts that involve this particular residue,

\begin{equation*} R=\frac{2}{n(n-1)}\sum ^n_{i=1,j=i+1}p\left(\mathbf {c}^i,\mathbf {c}^j\right). \end{equation*}

Finally, to quantify the change of contacts each intersubunit contact cluster experiences during rotation, the restriction score of all residues comprising the cluster was averaged.

### Collective motion analysis of H34 and S15

To obtain the dominant modes of motion of the flexible 23S rRNA helix H34 and protein S15, we carried out a principal component analysis (PCA) (68). After rigid-body fitting to the base of helix H34 using all atoms of the 23S nucleotides 700–702 and 730–732, first, all atoms of H34 (nucleotides 703–729) and S15 were extracted from the trajectories corresponding to all states. Next, these extracted trajectories were concatenated and the atomic displacement covariance matrix was calculated for atoms of H34 and of S15 separately. Finally, the trajectories were projected onto the first eigenvector of each covariance matrix. This projection then describes the progression along the most dominant mode of motion.

To capture the motion of the H34 tip relative to S15, the trajectories were rigid-body fitted to all S15 atoms and then all atoms from H34 nucleotides 714–716 were extracted. Here, the trajectory of each state was then projected onto the first and second eigenvector of the covariance matrix obtained from the concatenated trajectories.

## RESULTS AND DISCUSSION

### Intersubunit contact clusters

Throughout translation, the ribosomal subunits are associated by a complex network of non-covalent interactions across the intersubunit surface. Due to the large-scale body rotation and head swiveling (Figure 1b), different residues are involved in these interactions along the tRNA translocation pathway. To describe the dynamic interaction network, contacting residues were extracted from the 100-ns trajectories of 13 intermediate states of spontaneous translocation (25) which cover the whole range of body rotation (−2.7 to 16.3°) with all intermediate angles (Figure 1b). The method used for identification of contacting residues was shown earlier to accurately predict their conservation patterns (25). The residues that are linked by stable contacts were grouped into clusters (see ‘Materials and Methods’ section). Figure 1c shows the resulting 15 intersubunit contact clusters, where all residues involved in contacts with an occupancy of at least 50% are depicted. The occupancy of each contact in each state, as observed in the trajectories, is shown in Supplementary Tables S1–S25. For all previously defined bridges (29,37,40), corresponding residues were found in our intersubunit contact clusters (compare Table 1).

**Table 1.** Intersubunit contact cluster definitions

cluster	bridge(s)	30S residues	50S residues
1	B3,5,8	16S (337–340, 1418–1423, 1471–1472, 1483–1485)	23S (1768, 1947–1950, 1958–1961), L14 (13–14, 17–18, 47–49, 51, 54, 97–98, 100)
2	B2a,b	16S (1406–1410, 1492–1497, 1516–1517)	23S (1911–1917, 1919–1921, 1931–1932)
3	B4	16S (762–763), S15 (35, 39, 42-43, 46, 52, 55–56, 59, 62-63, 87–88)	23S (711–717)
4	B1a,b	S13 (22, 57, 59–60, 62–64, 66–67, 69–71, 73–74, 76–81, 91–92), S19 (28, 47, 55, 57–60, 62–65, 68, 75, 80)	23S (883–884, 887–889), L5 (107–114, 116, 133, 136, 146–148)
5	B8	16S (158–160, 341–348)	L14 (105, 108, 113–114, 116–123) L19 (35–38)
6	B7b	16S (710–713), S6 (13–14, 24, 73, 76–77, 79–82)	L2 (119–125, 129, 132–138, 162, 164–166, 174, 191, 268), L9 (86, 89, 123)
7	B5,6	16S (1429–1433, 1464–1465, 1468)	23S (1703–1704, 1751), L19 (65, 103–105, 108)
8		16S (683), S7 (110, 130, 135, 141–142, 148), S11 (12–13, 37, 74–75)	23S (2114–2116, 2140–2147, 2166–2167), L9 (125)
9	B5	16S (1473–1476)	23S (1689–1690, 1700–1702)
10	B6	16S (1439–1443, 1461–1463)	L19 (64, 86–87, 110–114)
11	B7a,b	16S (679–682, 702–703, 776), S6 (53)	23S (1846–1848, 1895, 2098–2100, 2191–2193), L2 (167, 172, 180, 182, 267)
12	B2c,7b	16S (772–775, 808)	23S (1820), L2 (1, 4, 160, 176, 200–201)
13	B1b	S13 (1–3, 5–9, 47, 49, 56, 65)	L5 (135, 138, 141–145)
14	B2b	16S (783–784)	23S (1835–1837)
15	B2c	16S (899–900)	23S (1693, 1830–1832)

For each cluster, the corresponding bridge name(s) (37), the 30S and 50S residues involved in contacts, with an occupancy more than 50% in at least one state, are shown.

The length of the simulations, 100 ns per state, does not allow to capture the full dynamics the subunit interface undergoes in the translocation intermediates. To estimate the influence of this limitation, we compared the contact pattern observed in a second independent 100-ns simulation of the pre1a state with the contact patterns obtained from all other simulations. Although the contact patterns from the two pre1a simulations are not identical (83% overlap of stable contacts with an occupany above 75%), as expected from the short time scales, they are more similar to each other than to the patterns from all other simulations (52 ± 9% mean overlap, see Supplementary Methods, Supplementary Figure S3). This observation suggests that the simulations sufficiently capture the differences in contacts between the states.

**Figure 3.**
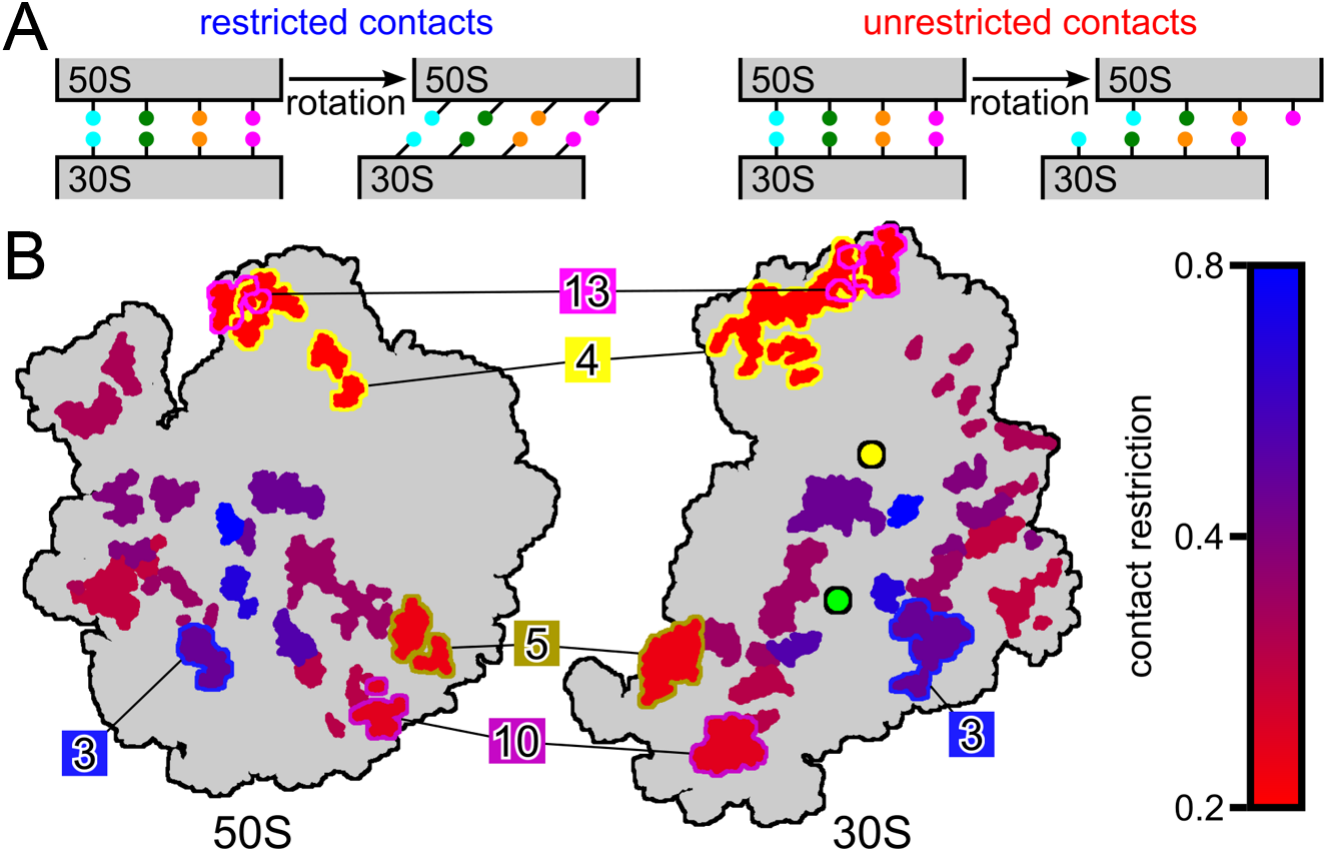
Changes of intersubunit contacts during translocation. (**A**) Schematic of two mechanisms to maintain intersubunit interaction despite rotation. (**B**) The contact restriction is color-coded for each intersubunit contact cluster. Clusters 3, 4, 5, 10 and 13 are highlighted. Pivot points are indicated for head (yellow circle) and body rotation (green circle), pivot point positions taken from Bock *et al*. (25).

The L1 stalk (H78), which facilitates tRNA translocation (5,21,22,25,26,69), contacts 30S proteins S7 and S11 of the 30S head and body domains, respectively (Figure 1c). The contacting residues (cluster 8) were suggested to link intersubunit rotation to L1-stalk closing and tRNA hybrid state formation (25). This coupling of 30S rotation to L1-stalk closing and P-site tRNA dynamics was recently confirmed by a smFRET study (26). The cluster 8 residues have not been previously assigned to any intersubunit bridge, probably because the L1 stalk is very flexible which renders it difficult to resolve its structure and leads to mostly transient contacts that differ for different translocation intermediates (Supplementary Table S8).

Intersubunit contact clusters 4 and 13 connect the 50S central protuberance (H38 and L5, see Figure 1a and c) with the 30S head domain (S13 and S19) which corresponds to bridges B1a and B1b (37). All remaining clusters connect the 50S and the 30S body domain. In particular, clusters 1, 2, 7, 9 and 10 involve nucleotides from the central 16S rRNA helix h44 on the 30S subunit, almost covering all h44 nucleotides pointing toward the subunit interface (compare Figure 1a and c). The h44 nucleotides A1418 and A1483 which belong to cluster 1 (bridge B3) close to the 30S body rotation pivot point (Figure 1b and c) were found to have the same conformation in non-rotated (−1.7° body rotation) and rotated (8.4°) ribosomes (11,31). In our simulations, these nucleotides are involved in stable contacts in all of the translocation intermediates (−2.7° to 16.3° body rotation, Supplementary Table S1), underscoring their crucial role as an axis of rotation.

Contact cluster 2 contains 23S rRNA helix H69 nucleotides corresponding to the B2 bridges which are central to many processes of translation (11,33,54,55). Dimethyl sulfate modifications of H69 nucleotides A1912 and A1918 strongly interfere with 70S formation (43) suggesting that they are important for subunit binding. Indeed, nucleotides A1912 and A1919 (next to A1918) were found to form stable contacts with h44 nucleotides in all intermediate states (Supplementary Table S2).

Close to h44 on the periphery of rotation, h8 and h14 are involved in contacts with proteins L14 and L19 (clusters 1 and 5). Cluster 3 consists of the tip of helix H34, which protrudes from the 50S subunit into a cavity formed by S15 and h20 of the 30S subunit. Further, clusters 6, 11 and 12 connect H68, H76 and L2 (50S) with helices h23 and h24 and protein S6 (30S). Finally, clusters 14 and 15 involve central RNA–RNA contacts corresponding to bridges B2b and B2c.

Upon hybrid state formation, which is accompanied by intersubunit rotation, 16S rRNA nucleotide A702 (cluster 11, bridge B7a) becomes solvent exposed (9,11,70,71). This is reflected by the observed loss of stable contacts between A702 and H68 nucleotides when going from early to late pre-states (Supplementary Table S11, pre3–pre5b). In the late pre-states, A702 forms more transient contacts with H76 residues, rendering it accessible to chemical probes (70). Finally, the A702 contacts to the H68 residues are restored when moving to post states.

Due to the relative rotation of the subunits, the 30S parts of the contact clusters move relative to their 50S counterparts, especially in the periphery of the subunit interface. This shift was previously observed in particular for the peripheral B1a/b bridges (clusters 4 and 13) (4,5,11,16,29,33,37,38,48). From our simulations, the largest relative shift of the 30S part relative to the 50S part was indeed found for clusters 4 and 13, where the centers of mass are shifted by more than 35 Å upon rotation.

In summary, the intersubunit contacts found in our simulations further extend the picture of intersubunit bridges by including all the different intermediate rotational states as well as the dynamics of the contacts. The relative shifts of the intersubunit contact clusters lead to the question of how the interaction network can compensate this shift to maintain a similar subunit affinity for different rotation angles. Do all contact clusters markedly contribute to the intersubunit affinity or is the affinity dominated by interactions of the central clusters, which are not subject to large shifts, as was previously suggested (37)?

### Intersubunit enthalpies during translocation

During large-scale intersubunit rotations, the intersubunit contact network undergoes substantial and complex dynamics, including breaking and formation of contacts. We now address the question of how the binding free energies of the contacts are balanced to maintain the overall affinity at remarkably constant level, a prerequisite for rapid rotation.

Here, we estimate the contribution of each intersubunit contact cluster to the stability of the 70S complex by calculating the interaction enthalpy between 30S and 50S residues of the cluster. Interaction enthalpy is estimated from the sum of electrostatic and van-der-Waals interactions. For each translocation intermediate and cluster, the interaction enthalpy was averaged over all 50 000 snapshots of the corresponding trajectory (Figure 1d).

Strong and steady enthalpic interactions were seen for clusters 1–7, providing the basis of intersubunit interaction (Figure 1d, left panel). Interactions of clusters were considered strong when their average contribution to the overall intersubunit enthalpy was more the 5% on average. The remaining clusters contribute to a lesser extent and show a higher variation of enthalpy in the different states (right panel upper part).

The central RNA–RNA bridges (B2a/b/c, B3), which are not subject to large shifts of their 30S parts relative to their 50S counterparts due to rotation, were suggested to be responsible for maintaining 70S stability (37) and to serve as anchoring patches for the rotations (42). Indeed, we observe strong interactions for the corresponding clusters 1 and 2 in all translocation intermediates. But, surprisingly, also clusters on the periphery, e.g. clusters 3–7, which consist of RNA and protein residues, interact strongly in all states, despite being subject to large shifts.

The intersubunit rotation shifts the 30S relative to the 50S parts of several clusters (clusters 1, 4 ,5 ,6 ,7, 10, 11 and 13) such that they interact with neighboring clusters (Figure 1d, right panel lower part). In the early pre-states (pre1a–pre2) and the post-states, the 50S part of cluster 4 interacts with the 30S part of cluster 13 (Figure 1c, lower part). Upon rotation, these contacts are lost and contacts between the 50S part of cluster 13 and the 30S part of cluster 4 are formed in the late pre-states. Overall, this leads to a strong and relatively constant contribution from these two most peripheral clusters.

The large contribution of contact clusters on the periphery of intersubunit rotation to the overall interaction enthalpy suggests that these interactions are crucial for the 70S stability. These large contributions would explain why mutations of peripheral residues decrease subunit association (43,44,47,49,72).

To check if the interaction enthalpy of a cluster is a reasonable measure of its relative contribution to 70S stability, we calculated the conformational entropy of the cluster residues using Schlitter's formula (73) (see Supplementary Methods). Indeed, the interaction enthalpy and a free-energy estimate which contains the interaction enthalpy and the conformational entropy are highly correlated (correlation coefficient 0.82). Note that solvent contributions to enthalpy and entropy were not considered in this free-energy estimate. Previously, a high correlation of the interaction enthalpy and the free energy which included solvent interactions was found for the L1 stalk–tRNA interaction (25). We do not claim that the interaction enthalpies approximate the free energy. However, the high correlation between the interaction enthalpy and the free-energy estimate for the contact clusters indicates that small enthalpy corresponds to small free energy and large enthalpy corresponds to large free energy which is sufficient for our conclusions.

### tRNAs contribute to the intersubunit enthalpy

Apart from direct 30S–50S interactions, also tRNAs contribute to the intersubunit affinity, as suggested by an increase in 70S complex stability when tRNAs are bound to the ribosome (32,60). The schematic in Figure 2a depicts, besides direct 30S–50S interactions (light red), all interactions between the subunits that are mediated by the tRNAs: tRNA–50S (pink), tRNA–30S (yellow), tRNA–mRNA (blue) and mRNA–30S (green). To estimate the contributions of the tRNAs to 70S stability, for each of these interactions and each state we calculated average interaction enthalpies (Figure 2b). The weakest interactions between the subunits via the tRNAs determine the contribution to the overall subunit interaction enthalpy. In our simulations, tRNAs are (enthalpically) more strongly bound to the 50S subunit (Figure 2b, pink) than to the 30S subunit (yellow) and the mRNA (blue), indicating that the tRNAs would remain bound to 50S subunit when pulling the subunits apart. In addition, the mRNA is bound more strongly to the 30S subunit (green) than to the tRNAs (blue). Consequently, the tRNA–30S (yellow) and the tRNA–mRNA interactions (blue) are the weakest interactions that connect the subunits via the tRNAs. This result suggests that only the tRNA–30S and tRNA–mRNA interactions are critical for the contribution of tRNAs to 70S complex stability. Notably, the combined tRNA–30S and tRNA–mRNA interaction is strongest when the anticodon stem-loop of the tRNA is in the 30S P site (tRNA^Val^: post states; tRNA^fMet^: pre-states) and weaker when it is bound to either the 30S A or 30S E site (Figure 2c, magenta and green). This interaction results in an overall rather constant intersubunit interaction enthalpy contribution of both tRNAs.

After tRNA translocation, the E-site tRNA first dissociates from the the 30S subunit assuming a position where it interacts mainly with the L1 stalk (L1 site) (24), before fully dissociating from the ribosome. Notably, when the tRNA^fMet^ moves from the E site toward the L1 site (states post1–post3b), the interaction enthalpies with the 50S and the 30S subunit gradually decrease from state to state (Figure 2b). This gradual decrease suggests that the affinity of the tRNA with the ribosome is not overcome in a single step, but that the affinity is reduced state by state thereby facilitating tRNA dissociation.

The comparison of the contributions of tRNAs and intersubunit contact clusters to the overall intersubunit interaction enthalpy (Figure 2c) suggest that the 70S stability is markedly increased by the binding of a tRNA in the P-site. On the basis of the interaction enthalpies alone, one would expect that the addition of an A-site tRNA to a ribosome in complex with a P-site tRNA would not markedly increase the subunit affinity. Notably, ribosomes which lack one of the strongest interaction clusters (cluster 2, bridge B2a) due to deletion of H69, still support EF-G dependent translocation at wild-type rates, but the subunits do not associate in absence of tRNAs (45). This finding is in line with our observation that the tRNAs contribute more to the enthalpic interaction between the subunits than cluster 2, such that the tRNAs can compensate for the loss of cluster 2 interactions.

Despite the large variation of interaction enthalpies of individual clusters and tRNAs, the sum of enthalpic contributions to intersubunit binding is similar in all states when considering the fluctuations observed in the states (Figure 2c, black bars). This similarity suggests that there are no large barriers induced by enthalpic interactions between the subunits, a prerequisite for rapid rotation.

### Contact restriction decreases with distance from pivot points

The remarkably steady interactions observed for intersubunit contact clusters on the periphery despite their large-scale relative shifts can be realized by two different mechanisms. First, residue–residue contacts could be maintained and the shift of 30S part relative to the 50S part of the cluster would be compensated by local structural deformations (Figure 3a, restricted). Second, residues could change their contact partner upon rotation (Figure 3a, unrestricted).

To examine which of the two mechanisms is applied, we calculated the level of contact restriction for each cluster (see ‘Materials and Methods’ section, Figure 3b, color-coded). Contact clusters close to the pivot points of head and body rotation generally have more restricted contacts, showing that the relative shift of 30S against 50S parts is mostly compensated by local deformations. In contrast, the further the cluster is away from the pivot points, the less restricted the corresponding contacts, with lowest restriction for clusters 4, 5, 10 and 13. Cluster 3, in contrast, shows more restricted contacts than other clusters at similar distances to the pivot points. In summary, steady interactions are achieved by changing contact partners where large-scale relative shifts make it necessary. These results suggest that the composition of contacting residues has evolved, not only to stabilize contacts close to the axes of rotation, but also to form a contact network that adapts to the rotation to maintain a strong and steady overall intersubunit interaction.

### Coulomb interactions stabilize 30S head bridges

The head of the 30S subunit, which rotates independently of the 30S body during intersubunit rotation (Figure 1b) (29–31), is connected to the 50S subunit by contact clusters 4 and 13 which show the lowest contact restriction. These clusters connect 30S proteins S13 and S19 with the central protuberance composed of 23S rRNA helix H38 and protein L5, corresponding to bridges B1a and B1b, respectively. S13 deficient ribosomes have defects in subunit joining and show increased translocation rates (49,72).

The combination of head swiveling and body rotation angles determines a specific positioning of L5 and H38 relative to S13 and S19. We compared the L5-S13 and L5-S19 contacts present in the simulations to those obtained from X-ray (11,29,48) and cryo-EM (16) structures of *Escherichia coli* ribosomes with different degrees of head swiveling and body rotation (compare Supplementary Table S26). Contacts involving H38 (bridge B1a) could not be compared to the X-ray structures since H38 is not resolved. The rotation angles of the X-ray structures were taken from Mohan *et al*. (31). The structure of a ribosome in complex with RRF and a tRNA in a P/E hybrid state (11) shows a 8.4° body rotation and a 4.8° head swiveling. This structure shares eight intersubunit contacts of L5 with our pre4 state simulation which undergoes body rotation between 8.2 and 11.2° and head swiveling between −3.6 and 5.1° (Supplementary Table S26). The overlap in rotation angles and contacts along with the low mutual rmsd of 4.7 Å (25) shows that both the overall conformation and the local intersubunit contacts are similar. Notably, the lower body rotation (−2.3°) but higher head swiveling (16.4°) seen in a structure of a vacant ribosome (29) places S13 and S19 in similar positions relative to L5 resulting in six intersubunit L5 contacts shared with the pre4 simulation. A structure of the vacant ribosome in complex with RF3 (48) has a similar head swiveling (16.3°), but higher body rotation (6.8°). Here, the head swiveling does not compensate for the body rotation such that this structure shares only one contact with the pre5a simulation which has higher body rotation (11.0 to 14.3°) and lower head swiveling (0.2 to 8.6°). Finally, a cryo-EM structure of ribosome in complex with EF-G in a pretranslocation state (16) shows the same head swiveling as the complex with RRF (11), but an increased body rotation (9.7°). The increased body rotation shifts the 30S head relative to the central protuberance, such that this structure shares five contacts (including contacts with H38) with the simulation of the pre5a state. High head swiveling angles (>10°) were only seen in structures of vacant ribosomes (9,29) and of ribosomes in complex with factors (EF-G, RF3, RRF) or antibiotics (10,13,15,17,20,48,74). As expected, these high head swiveling angles are not captured by our simulations which are based on cryo-EM maps of spontaneous tRNA translocation that do not contain states with high head swiveling angles (24). However, in our simulations, we observe the whole range of body rotation from −2.7 to 16.3° including all intermediate angles determining a wide range of contact patterns.

Based on two X-ray structures of non-rotated and rotated ribosomes, the relative movement of S13 and L5 (bridge B1b) has been proposed to resemble S13 moving as a rail in the groove of L5 (29). Here, the intermediate structures enable us to study the step-by-step change of the contact network.

**Figure 4.**
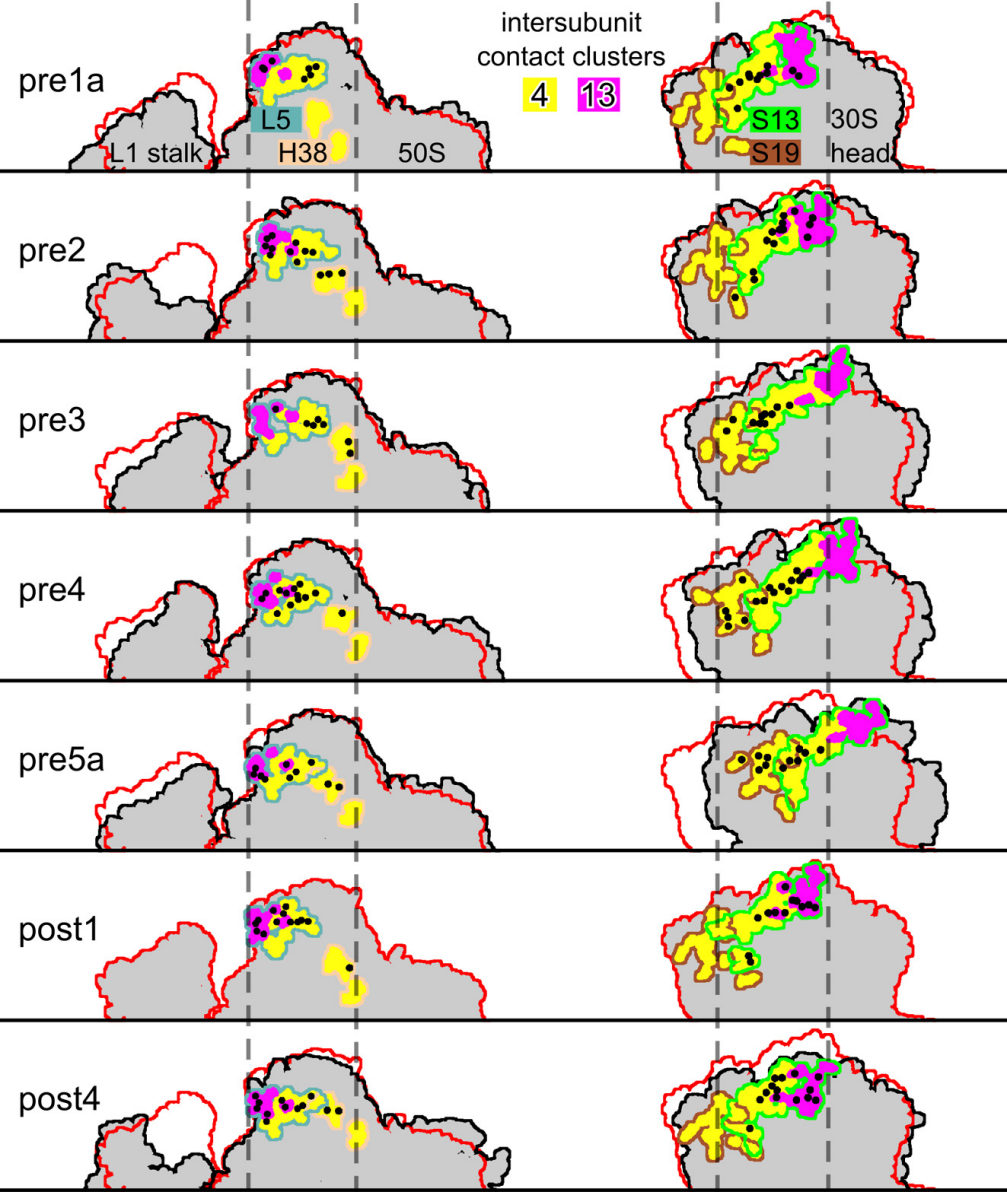
Contact network of clusters 4 (yellow) and 13 (pink). Residues forming intersubunit contacts with an occupancy larger than 50% are marked as black circles. The cluster residues belonging to L5, H38, S13 and S19 are identified by outlines. The outlines of 50S and 30S subunits are shown in black, with post1-state as reference (red). All structures are rigid-body fitted to the 50S residues (excluding the flexible L1 stalk) such that the 30S rotation can be seen.

Looking at the interacting residues of clusters 4 and 13 and their relative positions in the different intermediates (Figure 4, black circles), it can be seen that the set of contacting 50S residues changes little. In particular, three charged residues of L5 (R109, R111, D143) and C888 of H38 contact 30S head residues in at least 12 of 13 translocation intermediate states. In contrast, the set of contacting 30S residues changes markedly, whereas the positions of the contacting residues relative to the 50S subunit stay rather similar, as can be seen from the fact that they remain between the dotted lines in Figure 4. In the pre1 states, contacts of L5 and H38 residues with S13 residues are found (Supplementary Table S4). For the pre2 state, contacts are established between H38 and S19. In the pre4 state, L5 and S19 come into contact (R114-D63) and form several contacts in the highly rotated pre5b state (R111-M65, R111-E64, R113-E64, D146-S72). These contact patterns in the highly rotated late pre-states suggest to include H38 and S19 into the picture of a rail in a groove.

**Figure 5.**
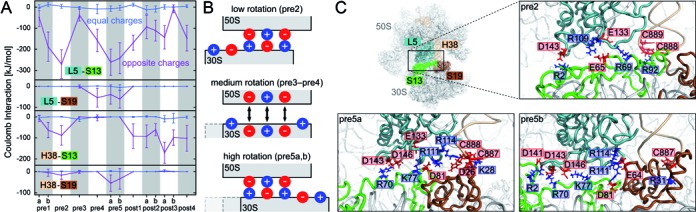
Stabilization of non-rotated and rotated states of clusters 4 and 13 (bridges B1a, B1b) by Coulomb interactions. (**A**) Coulomb interaction of L15 and H38 (50S subunit) with S13 and S19 (30S subunit). For each state and each pair of ribosomal parts, the average Coulomb interaction between charged residues carrying equal (blue lines) or opposite charges (magenta lines) is shown. Bars indicate the standard deviation. (**B**) Schematic of the Coulomb interaction patterns in different rotational states. (**C**) Close-up on the interaction site of L5, H38, S13 and S19 for pre2, pre5a and pre5b-states depicted in ribbon representation. Contact residues contributing at least 5% to the opposite charge interactions (a, magenta lines) are drawn as sticks. These residues are labeled and colored according to their charge (positive, blue; negative red).

The steady and strong interaction of clusters 4 and 13 in all translocation intermediates (Figure 1), enabled by changes of the contact partners (Figure 3), is remarkable. Frank *et al*. suggested that there are three rotational states with different S13–L5 interactions: a non-rotated state stabilized by opposite charges facing each other, an intermediate state destabilized by equal charges and a postulated highly rotated state again stabilized by opposite charges (75). This model is supported by rigid-body docking of proteins L5 and S13 into cryo-EM density maps of rotated and non-rotated ribosomes (76). To test this hypothesis, we analyzed the contribution of attractive and repulsive Coulomb interactions between the ribosomal parts forming the B1 bridges (L5, H38, S13, S19) to the strong interaction enthalpy that stabilizes different rotational states. To that aim, we calculated the average Coulomb interaction between all positively and negatively charged residues contributing to clusters 4 and 13 for each state (Figure 5a).

These Coulomb interactions were found to be markedly larger than hydrogen bond energies and Lennard-Jones energies, which describe van-der-Waals interaction and Pauli-repulsion (Supplementary Figure S4). This finding suggests that the energetics of clusters 4 and 13 are largely determined by the Coulomb interactions.

Attractive Coulomb interactions between residues with opposite charges were found between L5 and S13 as well as H38 and S13 (Figure 5a, magenta lines), in particular for low (pre1b and pre2) and for high rotation angles (pre5a and pre5b). Intersubunit rotation shifts the 30S head relative to the 50S central protuberance, such that additional attractive Coulomb interactions were seen for L5 and S19 as well as H38 and S19 for high rotation angles. In contrast, the repulsive Coulomb interactions between residues with equal charges are too small to influence the overall interaction enthalpy (Figure 5a, blue lines). The small repulsive interaction seen for intermediate rotations (pre3–pre4) suggests that the local conformation at the interface changes to avoid these unfavorable interactions, see schematic in Figure 5b. In summary, low and high rotation states are stabilized by attractive Coulomb interactions. Intermediate states are not unfavorable due to the presence of repulsive interactions, as hypothesized, but rather due to the lack of attractive interactions.

The increased translocation rate upon deletion of S13 was suggested to be caused by a destabilization of the pre-translocation states (49). Indeed, the contribution of S13 interaction with L5 and H38 for low and high rotation pre-translocation states is larger than in the post-translocation states, such that removal of S13 would lead to a relative destabilization of the pre-states. Further, for the high rotation states, interactions of L5 and H38 with S19 remain when S13 is not present, such that the high rotation states are favored over the low rotation states. The stabilization of the rotated state might additionally promote rapid tRNA translocation. This could be tested by mutation of S19 residue E64, which is in contact with L5 (R111, R114) in the highly rotated pre5b state (Supplementary Table S4), to an uncharged residue in addition to the deletion of S13. This mutation would destabilize the rotated state and would therefore reduce the translocation rate compared with the rate of the sole S13 deletion.

Figure 5c shows the charged residues involved in strong attractive Coulomb interactions in the low (pre2) and high (pre5a and pre5b) rotation states. In each of these three states, 5–7 strong attractive Coulomb interactions are seen (Figure 5c) that are presumably the main determinant for 30S head swiveling. Clusters 4 and 13 contain a large number of charged residues on the 30S subunit, S13 (R2, E49, R56, D57, E65, D67, R69, R70, E71, K77, R78, D81, R91, R92) and S19 (K28, D63, E64, R80), and on the 50S subunit, L5 (R109, R111, D112, R114, E133, D141, D143, K144, D146, R147) and H38 (G883, U884, U887, C888, C889). This abundance of charged residues allows for many different combinations of opposite charge interactions which in turn allows to stabilize different conformations. Consequently, only one residue (D143 of protein L5) is involved in contacts in all three states. In yeast ribosomes, mutations of protein L11 (corresponding to protein L5 in *E. coli*) which change side chain charges were shown to be either lethal or to lead to RNA structure changes (77), possibly by overstabilizing certain conformations. In contrast, mutations that neutralize charges did not show any effects, suggesting that there is a redundancy of charged residues in the B1 bridges (77).

Mutation of residue R2 of protein S13 to alanine (R3 in (26,72), which contributes to the strong interactions with L5 in the pre2 and the pre5b-state (Figure 5c), was shown to lead to a substantial growth defect, whereas the mutation of R92, which contacts H38 in the pre2 state, to glutamic acid had little effect (72). The different effects of these mutations can be explained by the relatively weak S13–H38 interactions that contribute less to the stability of the ribosome than the strong S13–L5 interactions. D81 (D82 in 26) of protein S13 strongly interacts with R111 (L5) in the pre5 states and thereby stabilizes the highly rotated state. Recently, the mutation of D81 to an alanine was indeed found to destabilize the rotated state (26). Using smFRET, this mutation was shown to decrease the rates of L1-stalk opening and of L1-stalk detaching from the P-site tRNA that are coupled to increasing intersubunit rotation. Further, R2 (S13) was mutated to alanine intending to destabilize the non-rotated state. Indeed, the rates of L1-stalk closing and contact formation with the P-site tRNA were increased for the R2A mutant. This finding underlines the importance of charged S13 residues in tuning the delicate balance between the low and high rotation states that are coupled to L1-stalk motion and consequently also to tRNA translocation.

### Bending of 23S rRNA helix H34 enables steady B4 bridge

The intersubunit contact cluster 3 (bridge B4) is located on the periphery of the subunit interface and consequently subject to a large-scale shift of its 30S relative to its 50S residues (Figure 1c). However, strong enthalpic interactions were found in all states (Figure 1d) suggesting that cluster 3 substantially contributes to the 70S stability (Figure 1c). Its high contact restriction (Figure 3b) raises the question of how contact partners can be maintained despite the large shift.

**Figure 6.**
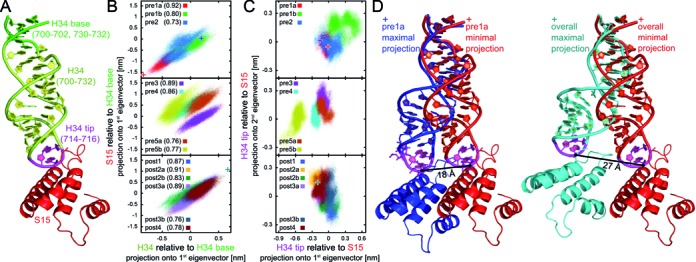
23S rRNA helix H34 follows the motion of 30S protein S15 to maintain the B4 bridge. (**A**) Ribbon representation of H34 and S15; regions of H34 used for PCA are indicated by color. (**B**) Projections of all trajectories onto the first eigenvectors calculated from a PCA of H34 and of S15 after rigid-body alignment of the H34 base. The numbers in brackets are the correlation coefficients of the projections in the corresponding states. The crosses denote structures with the minimal and maximal projections onto the first eigenvector of H34 (red cross: minimum of pre1a state and of all states; blue, cyan crosses, maximum of pre1a state and of all states, respectively). (**C**) Projections of all trajectories onto the first and second eigenvectors calculated from a PCA of H34 tip after rigid-body alignment of S15. Crosses refer to the same structures as in (B). (**D**) Structure of H34 and S15 corresponding to the minimal and maximal projection in the pre1a state (red and blue ribbons, compare crosses in (B)) and in all states (red and cyan ribbons, compare crosses in (B)).

The 50S part of cluster 3 consists of nucleotides 711–716 from H34 which is, besides H38 and H69, one of the prominent 23S rRNA helices protruding from the 50S surface (Figure 1a). These negatively charged nucleotides are in contact with S15 amino acids (Figure 6a), among them five positively charged arginines (R52, R62, R63, R87, R88) rendering strong electrostatic interactions possible (Table 1). Indeed, modification of A715, the central nucleotide of the H34 tip that is involved in contacts with S15 arginines in all states (Supplementary Table S3), was found to interfere with 70S complex formation (43). Further, H34 was seen in X-ray crystal structures to bend (7 Å movement of A715) upon a ∼9° body rotation allowing contact formation of A715 with S15 residues in both ribosome conformations (11). The 30S body rotation observed in our simulations spans a range from −2.7 to 16.3° which raises the question of how the dynamics of H34 enable steady contacts with S15 over this large range of rotation.

To describe the collective dynamics of H34, we extracted the dominant mode of H34 motion relative to the H34 base (Figure 6a), the first eigenvector resulting from a PCA. This mode of the H34 motion describes a bending motion of H34 in a direction tangential to the 30S body rotation. The dominant mode of S15 motion relative to the H34 base results from the 30S body rotation. Projection of a structure onto these modes measures the progression along the motion, e.g. the degree of bending. The projection of the MD trajectories, which correspond to the individual states, onto these modes is shown in Figure 6b. The H34 bending motion and the S15 motion are highly correlated (Figure 6b). This correlation indicates that H34 closely follows the relative movement of S15 in all of the states. The offset of the distributions of the projections seen in Figure 6b, e.g. for pre5a and pre5b, where H34 is bent to a different degree while S15 is at a similar relative position, suggests that the tip of H34 might form contacts with different regions of S15 in different states. Indeed, a PCA of the H34 tip relative to the position of S15 (Figure 6c) shows that it contacts S15 in slightly different regions in different states.

Strikingly, the bending of H34 seen in our simulations accounts for a motion of 18 Å in only the pre1a state and up to 27 Å in all of the translocation intermediate states (Figure 6d). The mean positions of A715 in the non-rotated pre1a and the highly rotated pre5b states deviate by only 9 Å which is only slightly larger than the 7 Å seen in the X-ray structures (11). The huge flexibility of H34 and the many charged residues of S15 allow to maintain the enthalpically strong contacts with S15, rendering cluster 3 one of the main anchors of intersubunit association.

## CONCLUSION

The two subunits of the ribosome are associated through intersubunit contacts during the entire process of translation (32). Rotation of the 30S head and body domains plays a crucial role in initiation (54,78), spontaneous and EF-G driven tRNA translocation (59), peptide release (48,71,79) and ribosome recycling (11,33).

Here, we have analyzed the dynamics and energetics of intersubunit contact clusters obtained from MD simulations of intermediate states of spontaneous tRNA translocation. Our approach is validated by the fact that residues found by X-ray crystallography (11,29,40,48) and cryo-EM (37) to contribute to intersubunit contacts were also identified in our simulations. Remarkably, intersubunit contact cluster 8 comprises residues of the L1 stalk and proteins S7 and S11 that have not been attributed to an intersubunit bridge before. These residues possibly stabilize the L1 stalk in the closed state (25).

To estimate the contributions of the individual intersubunit contact clusters to the 70S complex stability, interaction enthalpies for each cluster were calculated from the trajectories. The intersubunit contact clusters 1 and 2 contain bridges previously suggested to be mainly responsible for subunit association (37). These clusters indeed show a strong enthalpic interaction in all spontaneous-translocation intermediates. In addition, contact clusters 3–7, which are further away from the pivot points of rotation and therefore subject to larger shifts of their 30S parts relative to their 50S parts, show strong and steady interactions. Several mechanisms were identified that enable these strong interactions in all intersubunit rotation states.

The contact partners for clusters on the periphery of intersubunit rotation were found to change markedly for different rotational states, which enables strong interactions in all intermediate states. In particular, for contact clusters 4 and 13 corresponding to the B1 bridges, charged residues are located such that attractive Coulomb interactions between opposite charges stabilize low and high rotation states. In contrast, intermediate rotation states are destabilized by the lack of attractive Coulomb interactions. Interactions involving 30S protein S13, which is a part of contact clusters 4 and 13, were found to be stronger in the pre- than in the post-translocation states. This finding suggests that removal of S13 destabilizes pre- relative to post-translocation states, which rationalizes increased translocation rates observed for S13 depleted ribosomes (49).

In contrast to clusters 4 and 13, peripheral cluster 3 (bridge B4) has a relatively restricted contacts. Here, the flexibility of H34, which extends into the 30S subunit mainly contacting protein S15, allows H34 to follow the rotational movement maintaining strong interactions despite the large shifts. The complete distance covered by the tip of H34 in the simulations is much larger than the distance obtained from mean positions in individual states and static X-ray structures. This high flexibility of H34 allows it to follow the S15 movement during the complete body rotation and underlines the necessity to complement information derived from static structures with dynamics to understand the functional mechanisms of this molecular machine.

The tRNAs markedly contribute to the interaction enthalpy between the two subunits and have previously been shown to increase 70S complex stability (32,60). The observation that the joined contribution of both tRNAs to the intersubunit interaction enthalpy is almost constant, despite their different positions in the ribosome, suggests that the bridging of the subunits by the tRNAs does not introduce barriers hindering rotation and thereby contributes to rapid rotation. Notably, as the tRNA leaves the ribosome via the L1 site (24), the enthalpic interaction with the ribosome only gradually weakens. This gradual decrease suggests a step by step reduction of the tRNA affinity to the ribosome facilitating tRNA dissociation.

To estimate the contributions of tRNAs and direct intersubunit contacts to the stability of the ribosome, here we focused on studying the enthalpic interactions. Non-specific ions and water molecules were suggested to take part in intersubunit interactions (29,41,80). In particular, bridge B2c corresponding to cluster 12 was suggested to be purely ion stabilized (41). Thus the contribution of cluster 12, which showed only relatively weak direct enthalpic interactions, might be underestimated by our approach. The presented mechanisms for maintaining strong interactions during rotation involve strong Coulomb interactions of charged residues that are likely markedly stronger than indirect interactions.

Here, we describe mechanisms that, first, ensure the association of the enormous molecular machine while performing large-scale intersubunit rotation and, second, enable rapid rotation leading to efficient tRNA translocation. The total binding enthalpy remains rather constant, thereby on the one hand stabilizing the complex equally in all states and on the other hand enabling almost barrier-less rapid rotation.

In this work, we investigated the rotation around the main rotation axes of 30S head and body rotations. Recently, Budkevich *et al*. (19) found a rotation of the 30S subunit around its long axis in mammalian ribosomes. To what extent this motion is also seen in bacterial ribosomes and how it is facilitated by intersubunit contacts is still an open question that needs to be addressed. Here, interaction enthalpies between the subunits were studied which is sufficient for the qualitative analysis presented here. To understand how the presence of a single amino acid bound to the P-site tRNA hinders the intersubunit rotation that is spontaneously seen in ribosomes containing deacylated P-site tRNAs (5,34,81), requires to determine free energies, which will be the subject of future studies. The identification of paths along which information can be transmitted between subunits is a task that can also be tackled on the basis of a deeper understanding of the free-energy landscape of intersubunit rotation.

## SUPPLEMENTARY DATA

Supplementary Data are available at NAR Online.

SUPPLEMENTARY DATA
