# Supplementary material for: Dynamic contact network between ribosomal subunits enables rapid large-scale rotation during spontaneous translocation
Source: Nucleic Acids Res. 2015 Jun 24;43(14):6747–60. doi: 10.1093/nar/gkv649 (PMC4538834; doi:10.1093/nar/gkv649)
Supplement: SUPPLEMENTARY DATA [file supp_43_14_6747__index.html]

Dynamic contact network between ribosomal subunits enables rapid large-scale rotation during spontaneous translocation — Dynamic contact network between ribosomal subunits enables rapid large-scale rotation during spontaneous translocation — SUPPLEMENTARY DATA 

# Dynamic contact network between ribosomal subunits enables rapid large-scale rotation during spontaneous translocation

## SUPPLEMENTARY DATA

- SUPPLEMENTARY DATA
