# Supplementary material for: Dynamic contact network between ribosomal subunits enables rapid large-scale rotation during spontaneous translocation
Source: Nucleic Acids Res. 2015 Jun 24;43(14):6747–60. doi: 10.1093/nar/gkv649 (PMC4538834; doi:10.1093/nar/gkv649)
Supplement: SUPPLEMENTARY DATA [file supp_gkv649_nar-01413-r-2015-File013.pdf]

Supplementary Information

Dynamic Contact Network between Ribosomal Subunits enables Rapid Large-Scale Rotation during Spontaneous Translocation

Lars V. Bock, Christian Blau, Andrea C. Vaiana, and Helmut Grubmüller

Supplementary Methods

Choice of Occupancy Threshold for Clustering of Contacts

In the process of grouping contacts into intersubunit contact clusters (see Methods), in a first step, the contacts with an occupancy above a certain threshold were clustered (Supplementary Figure 1). This threshold was chosen on the basis of two criteria. First, the threshold should be in an interval where the number of clusters is independent of threshold variation. Second, the grouping of residues into clusters should depend as little as possible on the choice of the threshold.

To monitor the change of clustering of the residues, we applied the clustering protocol using thresholds ranging from 0.025 to 0.975. For each threshold, a $N \times N$ matrix $\mathbf{C}$ which describes the grouping of the N residues was constructed where $C_{ij} = 1$ when residues i and j are in the same cluster and $C_{ij} = 0$ otherwise. The difference of clustering $d(\mathbf{C^A}, \mathbf{C^B})$ between two matrices $\mathbf{C^A}$ and $\mathbf{C^B}$ was defined as

$$d(\mathbf{C^A}, \mathbf{C^B}) = \frac{\sum_{i,j}^{N} |C_{ij}^{A} - C_{ij}^{B}|}{N^2 - N}.$$

The sum of differences is normalized by the largest possible difference $N^2 - N$ between a matrix with all residues in one cluster and a matrix with each residue in a separate cluster. For each threshold, the difference between the matrix obtained using this threshold and the matrices using the two neighboring thresholds was calculated. The average of the two differences is shown in Supplementary Figure 2, along with the number of intersubunit contact clusters obtained for each threshold. Notably, for thresholds above 0.2 the number of clusters only fluctuates between 15 and 17 and also the difference of clustering is low. This result shows that the clustering protocol is robust with respect to the choice of the threshold. For further analysis, the clustering of contacts obtained with a threshold of 0.3 was chosen, because this threshold is in a region with constant numbers of clusters and with low difference of clustering to neighboring thresholds.

Contact Pattern Comparison

Contacts between the two ribosomal subunits were extracted from MD simulation of the ribosome in pre- (pre1a–pre5b) and post-translocation (post1–post4) states. To estimate how the trajectory length of 100 ns per state, influences the identification of intersubunit contacts, the sets of contacting residues extracted from different simulations was compared. To that aim, first, all the stable contacts with an occupancy larger than a certain threshold were recorded. The contact overlap between two simulation was defined as the ratio of the number of contacts found in both simulations by the number of contacts found in the first simulation. First, two sets of contacts obtained from two independent 100-ns simulations of the pre1a state were compared (Supplementary Figure 3, red line). Next, the contact overlaps between the first pre1a simulation and all other simulations (pre2–post4) was averaged (green line). The results suggests that the length of the simulations allows to capture differences in contact patterns between different states, despite the fact that the simulations are not fully converged.

All the contacts of 50S protein L5 with 30S proteins S13 and S19 (clusters 4 and 13, B1 bridges) extracted from our simulations were compared with contacts observed in X-ray structures of the ribosome in various states of rotation (1; 2; 3; 4). Two residues in the X-ray structures were considered to be in contact if the smallest distance between any of their atoms was below 5 Å. The contacts were compared with contacts from our simulations that have an occupancy above 50 %. The identified contacts common in X-ray structure and simulation are shown in Supplementary Table 26 for the state with the highest number of common contacts along with head and body rotation angles for the X-ray structure and for the simulation of the corresponding state.

Interaction Enthalpy and Conformational Entropy

To check if the enthalpy between 30S and 50S residues of an intersubunit contact cluster is a reasonable measure for its relative contribution to 70S stability, we compared the changes of enthalpy and conformational entropy along the dominant mode of motion for each cluster. Since some of the clusters interact with other clusters (compare Figure 1d) for the following analysis the interacting clusters were merged (1+5, 4+13, 6+11, 7+10).

To obtain the dominant mode of motion, a principal component analysis (PCA) (5) was performed on the heavy atoms of the cluster residues involved

in contacts with an occupancy of at least 50 % in any translocation intermediate simulation (compare Table 1). To that aim, from each frame and each trajectory (pre1a–post4), the coordinates of these atoms were extracted, rigid-body fitted and concatenated. The first eigenvector resulting from a PCA of this concatenated trajectory describes the dominant mode of motion.

To describe changes along the dominant mode of motion, the frames were sorted based on their projection onto the first eigenvector into 50 equally sized bins.

To measure the change of enthalpy along this motion, first, for each frame the interaction enthalpy H_i between the 50S and 30S residues was calculated as described in the Methods section. Then, for each bin the mean and standard deviation of the interaction enthalpy of all the frames included in this bin was calculated.

To estimate change of the conformational entropy S_c along the motion, for all the bins, Schlitter's formula (6) was applied to all frames included in this bin using the program g_anaeig from the GROMACS suite (7).

Finally, for each bin a free-energy estimate G_e, which does not contain solvent contributions, was calculated:

$$G_e = H_i - TS_c,$$

where $T = 300$ K is the temperature used in the simulations. A correlation coefficient of 0.82 is obtained when comparing G_e and H_i values for all bins of all clusters. This high correlation suggests that the enthalpy follows the same trend as the free energy and therefore is a good measure of the relative contributions of the different intersubunit contact clusters in the different states.

Supplementary Results

Contact Pattern Comparison

For all intersubunit contact clusters, Supplementary Tables 1–15 list the frequencies of residue-residue contacts between the 30S and the 50S subunit for each intermediate state. Supplementary Tables 16–25 list residue-residue contact frequencies of contacts between residues from different contact clusters. The gray-scale level of the cells indicates the frequency of atom-atom contacts corresponding to the residue pairs, white (0–12.5 %), light gray (12.5–25 %), dark gray (25–50 %), and black (50–100 %).

Supplementary Figures

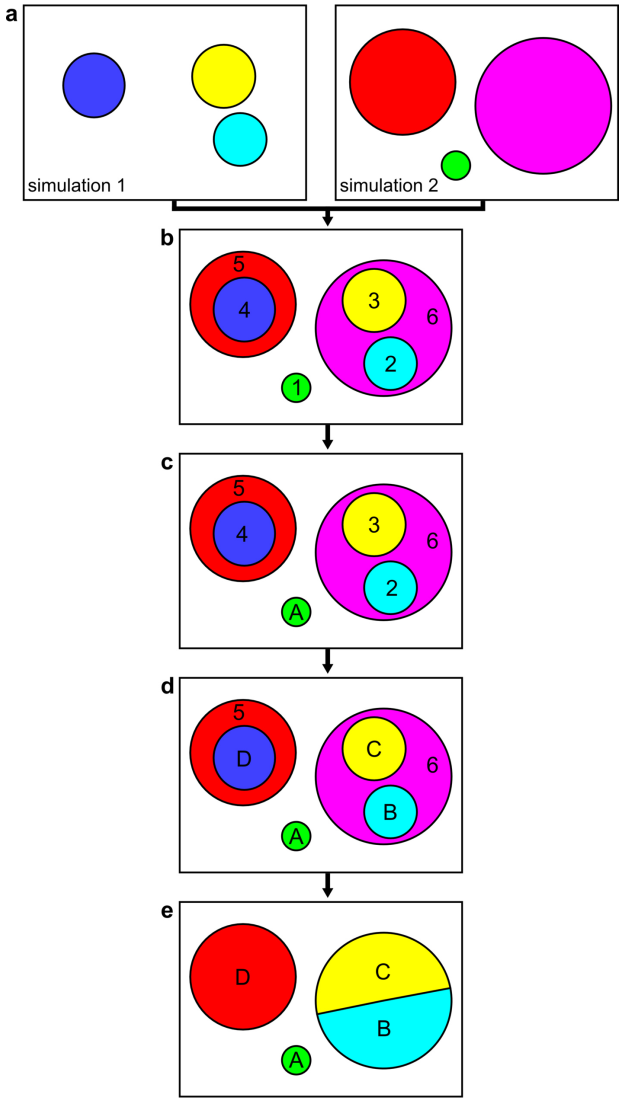

Supplementary Figure 1: Scheme of the clustering protocol applied to intersubunit contacts obtained from the simulations. (**a**) Clusters of contacts are represented by colored circles (from simulation 1: blue, orange, cyan; from simulation 2: red, magenta, green). (**b**) Clusters of different simulations were combined and sorted by size (small to large). (**c**) The smallest cluster was labeled new cluster A, and excluded from subsequent clustering. (**d**) With the remaining sorted clusters, this procedure was repeated until all clusters are assigned (clusters B, C, D). (**e**) Contacts from the remaining clusters were distributed to the closest new cluster, where distance was defined as the number of contacts which connect the residue to the cluster.

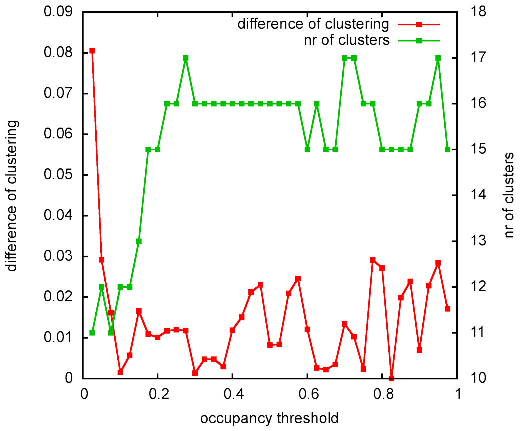

Supplementary Figure 2: Dependence of clustering of intersubunit contact on the threshold of contact occupancy. The difference of clustering is obtained by comparing the clustering at a specific threshold t with the clusterings at the two neighboring thresholds $t - 0.025$ and $t + 0.025$ (red line). For each threshold, the obtained number of contact clusters is shown (green line).

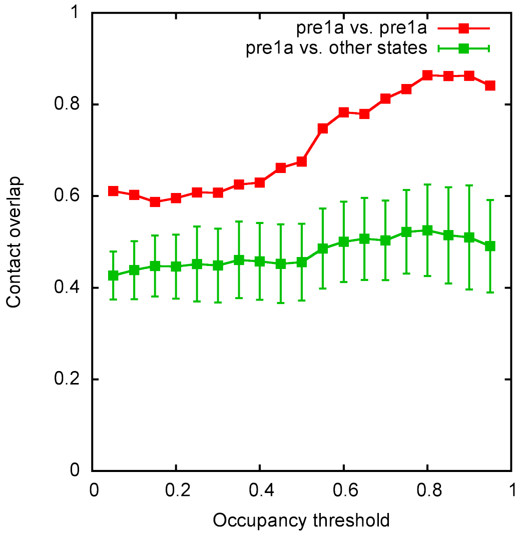

Supplementary Figure 3: Overlap of contacts contacts between two independent simulations of the pre1a state (red line) and between one pre1a simulation and the simulations of all other states (green, line, bars indicate standard deviation). The overlap was calculated for contacts with occupancies above different occupancy thresholds.

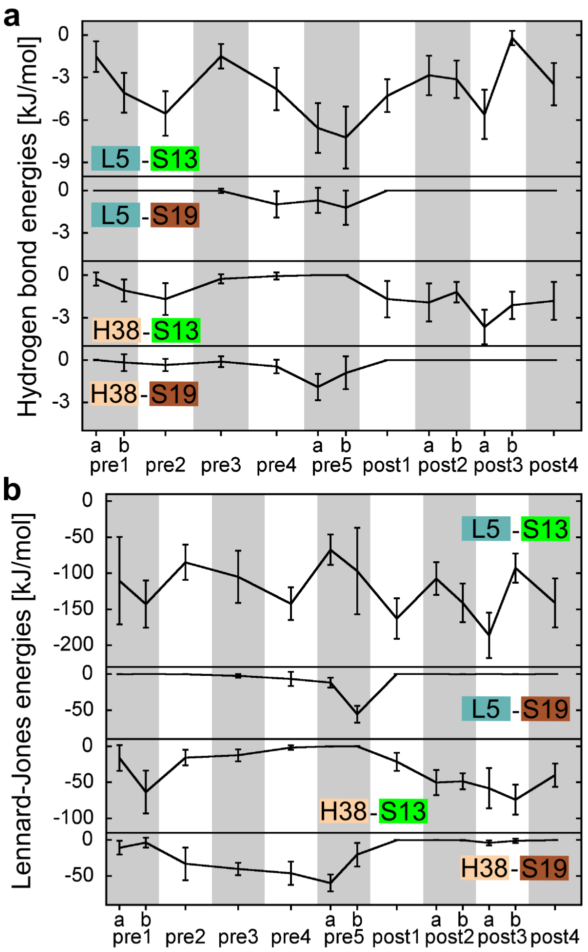

Supplementary Figure 4: Interactions of protein L5 and rRNA helix H38 with proteins S13 and S19 which contribute to intersubunit contact clusters 4 and 13 (B1 bridges). For each state and each pair of ribosomal parts the average hydrogen bond energies (**a**) and Lennard-Jones energies (**b**) are shown. Bars indicate standard deviation.

Supplementary Tables

Supplementary Table 1: Contacting residues for cluster 1.

30S residue	50S residue	pre							post					
		1a	1b	2	3	4	5a	5b	1	2a	2b	3a	3b	4
h44 A1418	H71 G1948													
h44 A1418	H71 G1959													
h44 G1419	H71 G1949													
h44 U1420	H71 G1950													
h44 A1483	H71 G1948													
h44 A1483	H71 G1959													
h44 C1484	H71 A1960													
h44 A1418	H71 C1958													
h44 G1422	L14 P48													
h44 A1483	H71 A1960													
h14 C339	L14 R98													
h44 G1421	L14 K54													
h44 A1483	H71 C1947													
h44 U1472	L14 R17													
h44 U1485	H71 C1961													
h14 C339	L14 N13													
h44 G1419	H71 G1950													
h44 C1484	H71 C1961													
h44 U1471	L14 R17													
h44 U1472	L14 R18													
h14 U340	L14 R98													
h14 U340	L14 N13													
h13 A338	L14 R98													
h44 U1420	L14 K54													
h44 A1418	H71 G1949													
h44 U1420	H71 G1949													
h14 C339	L14 S14													
h44 G1421	L14 P48													
h44 G1423	L14 R49													
h44 G1422	L14 I47													
h13 A338	L14 N13													
h44 G1419	H64 C1768													
h44 G1422	L14 R49													
h44 G1423	L14 P48													
h14 U340	L14 T97													
h44 G1422	L14 R17													
h13 A338	L14 F100													
h14 C339	L14 T97													
h13 A338	L14 S14													
h44 U1471	L14 R49													
h13 G337	L14 R98													
h14 C339	L14 K51													
h14 U340	L14 K51													
h14 C339	L14 R49													
h13 A338	L14 R49													
h13 A338	L14 R17													
h44 G1422	L14 K54													
h14 C339	L14 R17													
h44 C1484	H71 C1947													
h44 U1485	H71 A1960													
h44 C1484	H71 G1959													

Supplementary Table 2: Contacting residues for cluster 2.

30S residue	50S residue	pre							post					
		1a	1b	2	3	4	5a	5b	1	2a	2b	3a	3b	4
h44 G1494	H69 A1912													
h44 G1494	H69 A1913													
h44 U1495	H69 A1912													
h44 C1496	H69 A1919													
h44 A1408	H69 A1912													
h44 C1409	H69 A1916													
h45 G1517	H69 A1919													
h44 C1407	H69 A1912													
h44 U1495	H69 A1919													
h44 C1496	H69 C1920													
h44 C1407	H69 A1919													
h44 A1492	H69 A1913													
h44 A1493	H69 A1913													
h44 A1408	H69 A1916													
h44 C1409	H69 U1915													
h44 A1408	H69 A1913													
h44 C1409	H69 C1914													
h44 U1406	H69 A1919													
h45 G1517	H69 C1920													
h44 G1497	H69 C1920													
h45 G1516	H71 U1931													
h44 G1497	H69 G1921													
h44 A1408	H69 U1917													
h44 U1495	H69 A1913													
h44 U1495	H69 U1911													
h44 A1410	H69 C1914													
h44 A1408	H69 U1915													
h44 A1492	H69 C1914													
h44 G1494	H69 A1919													
h44 U1495	H69 C1920													
h45 G1517	H71 A1932													
h44 C1496	H69 G1921													
h44 A1408	H69 A1919													
h45 G1517	H71 U1931													
h44 C1409	H69 A1913													

Supplementary Table 3: Contacting residues for cluster 3.

30S residue	50S residue	pre							post					
		1a	1b	2	3	4	5a	5b	1	2a	2b	3a	3b	4
S15 V59	H34 A715													
S15 L55	H34 A715													
S15 R62	H34 A715													
S15 Q39	H34 A715													
S15 I35	H34 A715													
S15 R88	H34 A716													
S15 L56	H34 A715													
S15 R52	H34 A715													
S15 R63	H34 U714													
S15 R87	H34 U714													
S15 R87	H34 A716													
S15 R63	H34 A715													
S15 R87	H34 G713													
S15 Q39	H34 A716													
S15 R88	H34 A715													
S15 R88	H34 G712													
S15 R88	H34 G713													
S15 R87	H34 A715													
S15 R88	H34 U714													
h20 G763	H34 A716													
h20 U762	H34 A715													
S15 R87	H34 G712													
h20 G763	H34 A715													
S15 R87	H34 G711													
S15 R52	H34 A716													
S15 F42	H34 A715													
S15 F42	H34 A716													
S15 R88	H34 C717													
S15 L55	H34 A716													
S15 R52	H34 C717													
S15 A43	H34 A716													
S15 K46	H34 A716													
S15 K46	H34 C717													

Supplementary Table 4: Contacting residues for cluster 4.

30S residue	50S residue	pre 1a	1b	2	3	4	5a	5b	post 1	2a	2b	3a	3b	4
S13 R70	L5 D112													
S13 R69	L5 R111													
S13 R70	L5 F113													
S13 R70	L5 R111													
S13 G66	L5 D112													
S13 G66	L5 R111													
S13 R70	L5 R114													
S13 M74	L5 R114													
S13 K77	H38 C889													
S13 V59	L5 V107													
S13 V59	L5 I110													
S13 A60	L5 I110													
S13 A60	L5 F113													
S13 S73	H38 C888													
S13 R70	H38 C888													
S13 M74	H38 C888													
S13 V59	L5 F113													
S13 A60	L5 R114													
S13 F62	L5 R114													
S13 V63	L5 R114													
S13 R69	H38 C888													
S13 K77	H38 C888													
S13 R92	H38 C888													
S13 D67	L5 R111													
S13 R92	H38 C889													
S19 R80	H38 U887													
S19 R80	H38 C888													
S13 R69	L5 E133													
S13 R91	H38 C889													
S13 V64	L5 R111													
S13 A60	L5 R111													
S19 F60	H38 U887													
S13 I76	L5 R111													
S13 L79	L5 R111													
S13 M80	L5 D112													
S13 D81	L5 R114													
S13 K77	L5 R111													
S13 R78	L5 R111													
S13 M80	L5 R111													
S19 F60	H38 C888													
S13 D81	H38 C888													
S13 R78	L5 F113													
S13 M80	L5 F113													
S13 M80	L5 R114													
S19 T62	H38 C888													
S13 M80	H38 C888													
S13 K77	L5 E133													
S13 R70	L5 R109													
S13 G66	L5 I136													
S13 D67	L5 I136													
S13 R70	L5 I136													
S19 V57	H38 C888													
S19 P75	H38 C888													
S19 P58	H38 C888													
S13 D67	L5 R109													
S13 R69	L5 R147													
S19 D63	L5 R114													
S13 K77	L5 R109													
S13 D81	L5 R111													
S13 M74	L5 R109													
S19 Q55	H38 C888													
S13 R78	L5 D112													
S19 V59	H38 C888													
S19 T47	H38 C888													
S13 R70	L5 D146													
S19 K28	H38 U887													
S19 E64	L5 R114													
S13 S73	L5 D146													
S19 M65	L5 R111													
S19 E64	L5 R111													
S13 K77	L5 V148													
S13 E71	L5 D146													
S13 K77	L5 D146													
S19 H68	L5 R111													
S13 R91	H38 U887													
S13 Y22	L5 R111													
S13 R69	L5 R109													
S13 G66	L5 P108													
S13 R91	H38 C888													
S13 G66	L5 I110													

Continued Supplementary Table 4: Contacting residues for cluster 4.

30S residue	50S residue	pre 1a	1b	2	3	4	5a	5b	post 1	2a	2b	3a	3b	4
S13 S73	L5 R114													
S13 S73	L5 D112													
S13 D67	L5 R114													
S13 R91	H38 G883													
S13 R91	H38 U884													
S13 M74	H38 C889													
S13 Y22	L5 E133													
S13 D57	L5 R109													
S13 R70	L5 L116													
S13 R78	H38 U887													
S13 A60	L5 D112													

Supplementary Table 5: Contacting residues for cluster 5.

30S residue	50S residue	pre 1a	1b	2	3	4	5a	5b	post 1	2a	2b	3a	3b	4
h14 G346	L19 R38													
h14 G346	L19 K36													
h14 C345	L14 S117													
h14 C345	L14 K114													
h14 C345	L19 K36													
h14 C342	L14 L118													
h14 C341	L14 L118													
h14 C345	L14 P120													
h14 G347	L19 R38													
h14 C345	L14 M113													
h14 U343	L14 L118													
h14 G347	L14 L118													
h14 G347	L14 A119													
h14 G346	L14 E121													
h14 G346	L14 R108													
h14 G346	L14 R105													
h14 G347	L14 E121													
h14 G347	L14 P120													
h14 C345	L14 R108													
h8 G159	L19 R38													
h14 C345	L14 I116													
h14 C345	L19 S35													
h8 G158	L19 R38													
h14 U343	L14 S117													
h14 G347	L14 R105													
h8 G159	L19 K36													
h14 C342	L14 A119													
h8 A160	L19 K36													
h14 G347	L14 V122													
h14 C341	L14 A119													
h14 C345	L14 L118													
h8 G158	L19 K36													
h14 G346	L14 S117													
h8 G158	L19 K37													
h8 G159	L19 K37													
h14 A344	L19 K36													
h14 U343	L14 K114													
h14 C345	L19 R38													
h14 C345	L14 R105													
h14 C345	L14 V122													
h14 A344	L14 S117													
h14 G346	L14 L118													
h14 G347	L14 X123													
h14 C345	L14 E121													

Supplementary Table 6: Contacting residues for cluster 6.

30S	50S	pre							post					
residue	residue	1a	1b	2	3	4	5a	5b	1	2a	2b	3a	3b	4
S6 F80	L2 P135													
S6 D82	L2 R166													
S6 R24	L2 D120													
S6 F80	L2 A121													
S6 R79	L2 P135													
S6 F80	L2 L191													
S6 T76	L2 I123													
S6 F80	L2 I134													
S6 F80	L2 L129													
S6 F80	L2 N133													
S6 F80	L2 S138													
S6 D82	L2 N133													
S6 E73	L2 I123													
S6 R24	L2 V119													
S6 N81	L2 N133													
S6 D82	L2 R132													
h23 G713	L2 R174													
S6 D82	L2 P135													
h23 A712	L2 V164													
S6 T77	L2 I123													
h23 A712	L2 A165													
S6 Q14	L2 R132													
S6 F80	L2 I123													
h23 G711	L2 R268													
h23 A712	L2 R174													
h23 A712	L2 R268													
S6 N81	L2 P135													
S6 D82	L2 V136													
h23 G711	L2 V136													
h23 G713	L2 Q162													
h23 G711	L2 V164													
h23 G711	L2 A165													
h23 A712	L2 V136													
S6 E73	L2 K124													
h23 A712	L2 Q162													
h23 G711	L2 G137													
h23 G710	L2 V164													
S6 F80	L2 A122													
S6 F80	L2 K124													
h23 A712	L2 G137													
S6 F80	L2 P125													
h23 G710	L2 A165													
h23 G710	L2 V136													
S6 D13	L9 K89													
S6 R24	L9 D86													

Supplementary Table 7: Contacting residues for cluster 7.

30S	50S	pre							post					
residue	residue	1a	1b	2	3	4	5a	5b	1	2a	2b	3a	3b	4
h44 A1429	H62 G1703													
h44 U1464	L19 R108													
h44 A1431	L19 T103													
h44 A1429	H62 C1704													
h44 A1430	L19 T103													
h44 A1431	L19 G104													
h44 U1464	L19 K105													
h44 A1431	L19 N65													
h44 A1433	L19 N65													
h44 G1432	L19 T103													
h44 A1468	L19 N65													
h44 A1465	L19 K105													
h44 G1432	L19 N65													
h44 A1431	L19 K105													
h44 G1432	L19 K105													
h44 A1465	L19 R108													
h44 G1432	L19 G104													
h44 A1430	H62 C1704													
h44 U1464	H63 U1751													

Supplementary Table 8: Contacting residues for cluster 8.

30S	50S	pre							post					
residue	residue	1a	1b	2	3	4	5a	5b	1	2a	2b	3a	3b	4
S11 R12	H78 G2144													
S11 R12	H78 C2143													
S11 K13	H78 C2146													
S11 R12	H78 C2145													
S11 R12	H78 G2141													
S11 R12	H78 A2142													
S11 K13	H78 G2140													
S11 K74	H78 G2141													
S7 R142	H78 A2147													
S11 K74	H78 G2140													
S11 E75	H78 G2140													
S7 K130	H76 G2116													
S7 R142	H76 A2114													
S7 R110	H78 U2167													
S7 K130	H78 U2166													
S7 H141	H76 G2115													
S7 R142	H76 G2115													
S7 K148	H78 A2147													
S11 Q37	L9 T125													
S7 H141	H76 G2116													
S7 K135	H78 C2146													
S11 K74	H78 C2146													
S11 K13	H78 G2144													

Supplementary Table 9: Contacting residues for cluster 9.

30S	50S	pre							post					
residue	residue	1a	1b	2	3	4	5a	5b	1	2a	2b	3a	3b	4
h44 G1475	H62 A1689													
h44 G1475	H62 A1700													
h44 A1476	H62 A1690													
h44 A1476	H62 A1689													
h44 U1474	H62 A1700													
h44 U1474	H62 A1701													
h44 G1473	H62 G1702													
h44 U1474	H62 G1702													
h44 G1475	H62 A1701													

Supplementary Table 10: Contacting residues for cluster 10.

30S	50S	pre							post					
residue	residue	1a	1b	2	3	4	5a	5b	1	2a	2b	3a	3b	4
h44 A1441	L19 L113													
h44 G1442	L19 L113													
h44 A1441	L19 E111													
h44 C1462	L19 E111													
h44 C1462	L19 R87													
h44 G1461	L19 R87													
h44 G1442	L19 N114													
h44 G1442	L19 K86													
h44 G1442	L19 R87													
h44 A1441	L19 R112													
h44 A1441	L19 N114													
h44 A1441	L19 R87													
h44 A1441	L19 K86													
h44 U1440	L19 R87													
h44 U1440	L19 L113													
h44 C1443	L19 K86													
h44 U1463	L19 S64													
h44 C1462	L19 S64													
h44 G1439	L19 R87													
h44 G1461	L19 L113													
h44 C1443	L19 N114													
h44 G1442	L19 R112													
h44 A1441	L19 K110													
h44 U1463	L19 R87													

Supplementary Table 11: Contacting residues for cluster 11.

30S residue	50S residue	pre 1a	1b	2	3	4	5a	5b	post 1	2a	2b	3a	3b	4
h23 A702	H68 G1846													
h23 A702	H68 C1895													
h23 A702	H68 A1848													
h23 A702	H68 A1847													
h23 A681	L2 V267													
h23 C680	L2 V267													
h23 C680	L2 M180													
h23 A702	H76 U2099													
h23 A702	H76 G2100													
h23 A681	L2 T172													
h23 G682	L2 K182													
h23 A681	L2 K182													
h23 A681	L2 M180													
h23 A702	H76 U2192													
h23 A702	H76 U2098													
h23 A702	H76 A2191													
h23 G703	H76 U2098													
h23 A702	H76 G2193													
S6 K53	L2 D167													

Supplementary Table 12: Contacting residues for cluster 12.

30S residue	50S residue	pre 1a	1b	2	3	4	5a	5b	post 1	2a	2b	3a	3b	4
h24 G773	L2 M200													
h24 U772	L2 L201													
h24 G774	L2 R176													
h24 U772	L2 M200													
h24 G774	L2 Y160													
h24 G775	L2 R176													
h24 G773	L2 L201													
h24 G775	L2 Y160													
h24 G774	H66 U1820													
h24 G774	L2 M200													
h24 U772	L2 K4													
h24 U772	L2 A1													
h24 G773	L2 K4													
h24 C808	L2 A1													

Supplementary Table 13: Contacting residues for cluster 13.

30S residue	50S residue	pre 1a	1b	2	3	4	5a	5b	post 1	2a	2b	3a	3b	4
S13 R2	L5 D143													
S13 I8	L5 V145													
S13 R2	L5 Y142													
S13 E65	L5 I135													
S13 E65	L5 Y142													
S13 R2	L5 D141													
S13 I6	L5 K144													
S13 I8	L5 D143													
S13 I8	L5 Y142													
S13 P9	L5 D143													
S13 I8	L5 P138													
S13 I8	L5 K144													
S13 A1	L5 Y142													
S13 I3	L5 P138													
S13 E49	L5 P138													
S13 R56	L5 I135													
S13 P9	L5 Y142													
S13 L47	L5 Y142													
S13 L47	L5 D141													

Supplementary Table 14: Contacting residues for cluster 14.

30S residue	50S residue	pre 1a	1b	2	3	4	5a	5b	post 1	2a	2b	3a	3b	4
h24 A784	H68 C1837													
h24 C783	H68 C1836													
h24 A784	H68 C1836													
h24 C783	H68 G1835													

Supplementary Table 15: Contacting residues for cluster 15.

30S residue	50S residue	pre 1a	1b	2	3	4	5a	5b	post 1	2a	2b	3a	3b	4
h27 C899	H67 G1831													
h27 A900	H67 C1832													
h27 A900	H67 G1831													
h27 C899	H67 C1830													
h27 C899	H62 U1693													

Supplementary Table 16: Contacting residues for cluster 4 (30S) and cluster 13 (50S).

30S residue	50S residue	pre 1a	1b	2	3	4	5a	5b	post 1	2a	2b	3a	3b	4
S13 R69	L5 P138													
S13 G66	L5 Y142													
S13 D67	L5 Y142													
S13 R70	L5 I135													
S13 R70	L5 D143													
S13 R70	L5 K144													
S13 S73	L5 D143													

Supplementary Table 17: Contacting residues for cluster 5 (30S) and cluster 1 (50S).

30S residue	50S residue	pre 1a	1b	2	3	4	5a	5b	post 1	2a	2b	3a	3b	4
h14 C341	L14 R98													
h14 G347	L14 R98													
h14 G348	L14 R98													
h14 C342	L14 R98													

Supplementary Table 18: Contacting residues for cluster 8 (30S) and cluster 6 (50S).

30S residue	50S residue	pre 1a	1b	2	3	4	5a	5b	post 1	2a	2b	3a	3b	4
h23 G683	L9 R123													

Supplementary Table 19: Contacting residues for cluster 9 (30S) and cluster 1 (50S).

30S residue	50S residue	pre 1a	1b	2	3	4	5a	5b	post 1	2a	2b	3a	3b	4
h44 G1473	L14 R18													

Supplementary Table 20: Contacting residues for cluster 9 (30S) and cluster 7 (50S).

30S residue	50S residue	pre 1a	1b	2	3	4	5a	5b	post 1	2a	2b	3a	3b	4
h44 G1473	H62 G1703													

Supplementary Table 21: Contacting residues for cluster 10 (30S) and cluster 7 (50S).

30S residue	50S residue	pre 1a	1b	2	3	4	5a	5b	post 1	2a	2b	3a	3b	4
h44 U1463	L19 R108													
h44 U1463	L19 K105													

Supplementary Table 22: Contacting residues for cluster 11 (30S) and cluster 6 (50S).

30S residue	50S residue	pre 1a	1b	2	3	4	5a	5b	post 1	2a	2b	3a	3b	4
h24 G776	L2 R174													
h23 C680	L2 R268													
h23 C679	L2 R268													
h23 A681	L2 A165													
h23 A681	L2 R268													
h23 C680	L2 V164													
h23 A681	L2 V164													

Supplementary Table 23: Contacting residues for cluster 11 (30S) and cluster 12 (50S).

30S residue	50S residue	pre 1a	1b	2	3	4	5a	5b	post 1	2a	2b	3a	3b	4
h24 G776	L2 Y160													

Supplementary Table 24: Contacting residues for cluster 12 (30S) and cluster 6 (50S).

30S residue	50S residue	pre 1a	1b	2	3	4	5a	5b	post 1	2a	2b	3a	3b	4
h24 G775	L2 R174													

Supplementary Table 25: Contacting residues for cluster 13 (30S) and cluster 4 (50S).

30S residue	50S residue	pre 1a	1b	2	3	4	5a	5b	post 1	2a	2b	3a	3b	4
S13 E65	L5 R111													
S13 E65	L5 R109													
S13 I6	L5 R109													
S13 R2	L5 P108													
S13 I3	L5 V107													
S13 I3	L5 P108													
S13 G5	L5 R109													
S13 I8	L5 I136													
S13 I6	L5 R111													
S13 N7	L5 I136													
S13 R56	L5 P108													
S13 I6	L5 I136													
S13 I8	L5 R109													
S13 E65	L5 P108													
S13 R2	L5 I136													
S13 R56	L5 R109													
S13 I3	L5 I136													
S13 I6	L5 P108													
S13 P9	L5 I136													
S13 R2	L5 D146													

Supplementary Table 26: Comparison of the contacts found in the trajectories for intersubunit contact clusters 4 and 13 (B1 bridges) with contacts obtained from X-ray structures.

Reference	body rotation[a]	head swiveling[a]	state[b]	body rotation[c]	head swiveling[c]	common contact(s)
Dunkle et al. (2)	8.4°	4.8°	pre4	8.2° to 11.2°	−3.6° to 5.1°	L5 109 - S13 70, L5 111 - S13 77, L5 111 - S13 78, L5 114 - S19 63, L5 133 - S13 77, L5 135 - S13 70, L5 136 - S13 70, L5 142 - S13 66
Schuwirth et al. (1)	−2.3°	16.4°	pre4	8.2° to 11.2°	−3.6° to 5.1°	L5 109 - S13 70, L5 111 - S13 78, L5 114 - S19 63, L5 135 - S13 70, L5 136 - S13 70, L5 142 - S13 66
Zhou et al. (3)	16.3°	6.8°	pre5a	11.0° to 14.3°	0.2° to 8.6°	L5 144 - S13 70
Brilot et al. (4)	9.7°	4.8°	pre5a	11.0° to 14.3°	0.2° to 8.6°	L5 111 - S13 81, L5 143 - S13 70 H38 888 - S19 47, H38 888 - S19 58, H38 888 - S19 60

[a] angle taken from Mohan et al. (8)

[b] state for which the corresponding simulation showed the highest number of common contacts with the X-ray structure.

[c] range of angles observed in the simulation.

References

1. Schuwirth,B.S., Borovinskaya,M.A., Hau,C.W., Zhang,W., Vila-Sanjurjo,A., Holton,J.M., and Cate,J.H.D. (2005) Structures of the bacterial ribosome at 3.5 A resolution. *Science*, **310**, 827–834.

2. Dunkle,J.A., Wang,L., Feldman,M.B., Pulk,A., Chen,V.B., Kapral,G.J., Noeske,J., Richardson,J.S., Blanchard,S.C., and Cate,J.H.D. (2011) Structures of the bacterial ribosome in classical and hybrid states of tRNA binding. *Science*, **332**, 981–984.

3. Zhou,J., Lancaster,L., Trakhanov,S., and Noller,H.F. (2012) Crystal structure of release factor RF3 trapped in the GTP state on a rotated conformation of the ribosome. *RNA*, **18**, 230–240.

4. Brilot,A.F., Korostelev,A.A., Ermolenko,D.N., and Grigorieff,N. (2013) Structure of the ribosome with elongation factor G trapped in the pretranslocation state. *Proc. Natl. Acad. Sci. U.S.A.*, **110**, 20994–20999.

5. Amadei,A., Linssen,A.B., and Berendsen,H.J. (1993) Essential dynamics of proteins. *Proteins*, **17**, 412–425.

6. Schlitter,J. (1993) Estimation of absolute and relative entropies of macromolecules using the covariance matrix *Chemical Physics Letters*, **215**, 617–621.

7. Pronk,S., Páll,S., Schulz,R., Larsson,P., Bjelkmar,P., Apostolov,R., Shirts,M.R., Smith,J.C., Kasson,P.M., van derSpoel,D., Hess,B., and Lindahl,E. (2013) GROMACS 4.5: a high-throughput and highly parallel open source molecular simulation toolkit. *Bioinformatics*, **29**, 845–854.

8. Mohan,S., Donohue,J.P., and Noller,H.F. (2014) Molecular mechanics of 30S subunit head rotation. *Proc. Natl. Acad. Sci. U.S.A.*, **111**, 13325–13330.
